# Preference-based serial decisions are counterintuitively influenced by emotion regulation and conscientiousness

**DOI:** 10.1371/journal.pone.0222797

**Published:** 2019-10-04

**Authors:** Sangsup Yoon, Sewoong Lim, Jaehyung Kwon, Jerald D. Kralik, Jaeseung Jeong

**Affiliations:** Department of Bio and Brain Engineering, Korea Advanced Institute of Science and Technology (KAIST), Daejeon, Republic of Korea; Universiteit Leiden, NETHERLANDS

## Abstract

Our decisions have a temporally distributed order, and different choice orders (e.g., choosing preferred items first or last) can lead to vastly different experiences. We previously found two dominant strategies (favorite-first and favorite-last) in a preference-based serial choice setting (the ‘sushi problem’). However, it remains unclear why these two opposite behavioral patterns arise: i.e., the mechanisms underlying them. Here we developed a novel serial-choice task, using pictures based on attractiveness, to test for a possible shared mechanism with delay discounting, the ‘peak-end’ bias (i.e., preference for experienced sequences that end well), or working-memory capacity. We also collected psychological and clinical metric data on personality, depression, anxiety, and emotion regulation. We again found the two dominant selection strategies. However, the results of the delay, peak-end bias, and memory capacity tasks were not related to serial choice, while two key psychological metrics were: emotion regulation and conscientiousness (with agreeableness also marginally related). Favorite-first strategists actually regulated emotions better, suggesting better tolerance of negative outcomes. Whereas participants with more varied strategies across trials were more conscientious (and perhaps agreeable), suggesting that they were less willing to settle for a single, simpler strategy. Our findings clarify mechanisms underlying serial choice and show that it may reflect a unique ability to organize choices into sequences of events.

## Introduction

Whatever we do—e.g., eating a meal, listening to music, working our jobs—we ultimately choose actions one-by-one, which imposes a sequential order on our series of decisions. In the same vein, we also experience life events in a serial order. Thus, the way we make our choices and evaluate our past events lies in this sequential nature of experience, yet the factors underlying its influences are poorly understood.

Unlike some cases that have a clear optimal solution for the order of actions to reach goals (e.g., route planning, manufacturing products, various games and sports), there are other cases that have no clear optimal order. Nonetheless, even in these cases, people normally behave according to specific orders. For example, Jeong et al. (2014) found that when selecting among pieces of sushi during a meal, people generally fell into one of two categories: picking the best option first (favorite-first) or saving the best for last (favorite-last). These two opposite behavioral strategies in people lead to further intriguing questions about the underlying origins of the two preferences, especially since rhesus monkeys appear to strongly favor selecting the best items first, and yet when selecting among entire sequences previously experienced (i.e., a mixture of retrospective and prospective evaluation), they prefer the sequences with the best items last [1, 2, 3].

It is clear that a favorite-first strategy may relate to delay discounting, i.e., the propensity to discount the value of future (delayed) events. Indeed, since substantial evidence supports the prevalence of delay-discounting behavior not only for humans [4, 5, 6, 7] but for many other animals, including primates and rodents [8, 9], it would seem plausible that the favorite-first selection strategy may derive from the same underlying mechanisms.

In contrast, the favorite-last strategy may relate to the “peak-end rule”, which is a well-established psychological heuristic used to evaluate past experiences. Presumably, due to a lack of memory capacity, the most salient (peak) and most recent (end) events have greater impact on our overall evaluation of a sequence of experiences [10, 11, 12, 13]. Therefore, particularly in cases with lower risk of losing the best item if not consumed instantly, a favorite-last strategy might optimize overall satisfaction of a sequential experience [14, 15].

Thus, it is indeed possible that the two serial-choice strategies of selecting favorite first or last result from more general underlying mechanisms, such as discounting the future, simplifying assessments of the past, or general memory capacity. It also remains possible, however, that sequential choice behavior reflects a unique phenomenon related to organizing and managing sequential events. Moreover, given the relative lack of understanding about the important higher-level cognitive ability of humans to comprehend sequential events as components of a larger event complex [15], determining the actual underlying mechanisms of serial choice comprehension and behavior is necessary.

Therefore, the aim of the current study was to employ a novel serial-choice task to examine how people make serial choices among a set of items that have different preference rankings. Similar to the “sushi problem”, our task was designed to ask participants to choose each option one-by-one until all options were selected, with no particular selection strategy being necessarily better than the others, since all items should be chosen eventually [1]. Because food items such as sushi run the risk of satiation in longer studies, we sought a stimulus that was also a real and immediately consumable reward, which yet could also maintain effectiveness across multiple trials and sessions. Attractiveness of people in visual images is one of the few that can potentially achieve the effect, and prior evidence supports this [16–20]. Yet at the same time, the difference between genders toward visual attractive/sexual stimuli is quite dramatic (both behaviorally and neurally), and thus must be dealt with separately for male and female subjects [19–23]. Therefore, based on the need for real and immediately consumable reward sustainable across the study, we used sexually attractive images with male participants only (with the expectation that female participants would be examined in the future).

We confirmed the motivational incentive of the pictures via requiring reliability in their ratings and an effort task described below. Furthermore, to examine possible relationships between serial-choice behavior and the other tested phenomena, we were able to use the same picture stimuli in comparable peak-end, delay discounting, effort, and working memory tasks with the same participants. Finally, we collected several additional pieces of psychological metric data to both validate our novel picture-based tasks using other traditional measures, as well as to examine further possible effects underlying the serial-choice behavior. For example, delay discounting has a well-established relationship with the Beck Depression Inventory (BDI; depression) and the State-Trait Anxiety Inventory (STAI; anxiety) index [24, 25, 26], which is also supposed to be related to working-memory capacity [27]. Besides these metrics that measure problematic symptoms, we also collected the Big Five Inventory (BFI), and Emotion Regulation Questionnaire (ERQ) data to test whether personality and emotion regulation factors, respectively, also influence serial choice.

## Methods

### Participants

Eighty healthy male subjects (self-reported heterosexual, age: 25.49 ± 3.01 [mean ± standard deviation] years) participated in the study. Because the experiment was conducted across four separate days, with the first two for the Picture-Rating Task (see Experimental Procedures below), some participants did not complete particular tasks due to not returning. Eleven participants did not return on the third day, and thus were dropped from the study altogether. Another three participants did not return on the fourth day, and thus did not provide data for the Delay-Discounting Task and ERQ questionnaire. Another participant’s data for the Delay-Discounting Task was excluded by the screening criteria (catch trials) (see task description below).

To provide the strongest tests of the hypotheses, we used all data available for each task. The alternative would be to drop all data from participants who did not complete the entire study (i.e., across all four days, as well as pass the Delay-Discounting Task screening). In fact, we also conducted the analyses with only the latter (65 participants), and there were no meaningful differences compared with maintaining the largest data sets for each task. We therefore report the results with all possible data being used. Again, only male participants were recruited for the current study based on the need to use a real and immediately consumable reward sustainable across repeated experimental conditions, and since they show stronger motivation (both via behavioral and neural responses) to view pictures of the opposite sex [16–23]. All participants were right-handed and had normal or corrected to normal vision with no history of neurological or psychiatric abnormalities. The Institutional Review Board (IRB) of KAIST approved all experimental procedures for this study. Informed written consent was obtained from all participants.

### Experimental stimuli

As explained above, in order to validate our novel test paradigm with a sustainable, consumable reward, in the current study we first tested male participants with pictures of the opposite sex (with the expectation that female participants will be tested in the future). A total of 500 pictures of females were used for the experiment. All pictures were collected from Google image search (https://images.google.com/) with keywords “Asian”, “woman”, “girl”, “bikini”, though many had various types of clothing. We selected pictures containing only a single subject with a clearly visible face and eye gaze. We excluded pictures with texts, animals or any emotionally salient objects like food, weapons, or luxury items. Pictures that were small or blurry or had a clear expression of negative emotion or appeared to be younger than 19 years old were also excluded [18, 19].

### Experimental procedures

To test for reliability and generalizability across days, the experiment was conducted across four separate days with an average of one week between each day (mean (M) = 8.21, standard deviation (SD) = 3.94). Each day’s complete experimental session took approximately 90 (up to 110) minutes. On the 1st and 2nd day, participants performed the Picture-Rating Task, which measured the participant-specific preferences for the experimental stimuli (i.e., pictures of the women). Two different days separated by approximately a week provided a means to test for and obtain reliable ratings. From these rating results, we sorted the pictures into seven groups, determined by the participant-specific preference levels (see Picture-Rating Task below). The subsequent experimental tasks used this classified set of pictures for each participant. On the 3rd day, the participants performed the Serial-Choice, Sequence-Rating, and Effort Tasks. To mitigate potential boredom, the trials of the Serial-Choice and Sequence-Rating Tasks were mixed and presented in pseudo-random order in the same session. The Effort Task was randomly interleaved between each Serial-Choice / Sequence-Rating session. In the Sequence-Rating Task trials, there were two questions: (1) asking the participant to rate the sequence, and (2) asking them to remember the order of pictures (to measure working-memory performance). On the 4th day, the Delay-Discounting Task was conducted. The experimental sessions were implemented using MATLAB (MathWorks, Natick, MA).

Prior to the calculation of the correlation coefficients between measures, we checked the normality of the data using the Shapiro-Wilk test. Except for the Serial-Choice Task (whose data were clearly not normally distributed and should not be transformed), for those distributions found to be skewed from normal (p < .05; S2 Table) we used the log transformation to approximate normality. We also added a minimum constant (i.e., 0.0001) to all variables before the log transformations (to enable transformation of all results). The statistical analyses were performed using the SPSS Version 22.0 statistics software package.

### Picture-rating task

**Fig 1:**
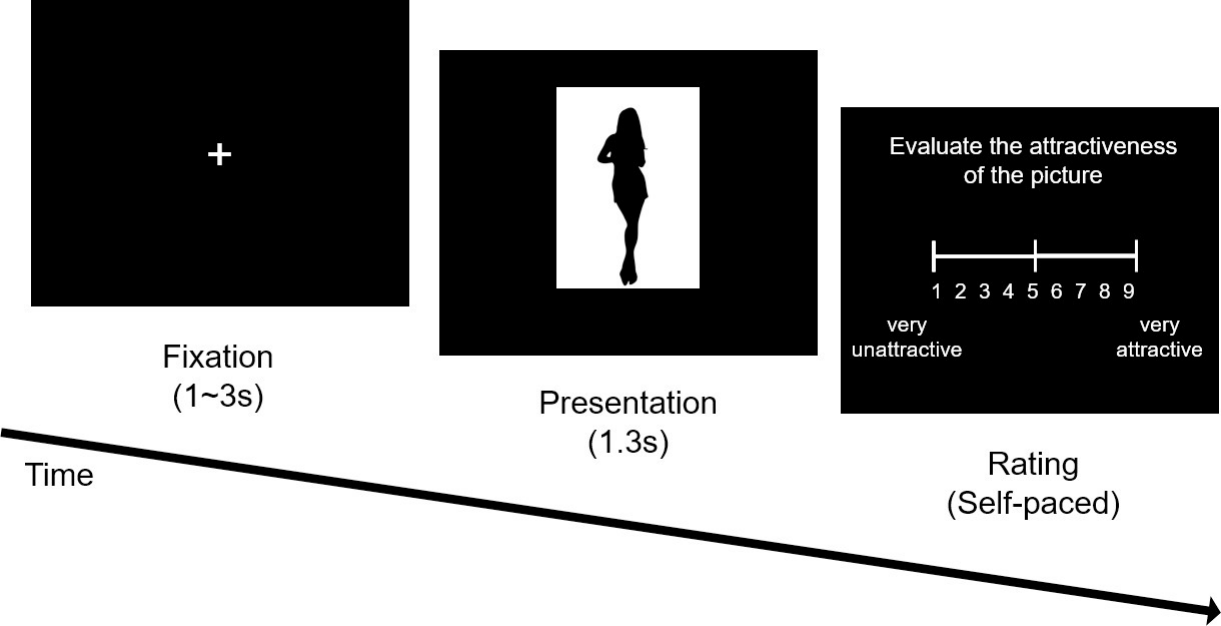
Illustration of the picture-rating task procedure.

The prepared set of 500 pictures was randomly divided into 10 subsets with 50 pictures, where each subset corresponded to each session of the Picture-Rating Task. Each session thus consisted of 50 trials, which first presented a picture for 1.3 seconds then asked the preference of that picture. The query “Evaluate the attractiveness of the picture.” (in Korean) was displayed on the screen with a horizontal 1~9 range scale bar below. When the participant pressed the number key on the keyboard, that number on the scale bar changed color from white to blue on the monitor. Then, when participants pressed the enter key, the evaluation was confirmed. Before pressing the enter key, the subject could freely change the active (blue) number on the scale bar without a time limit (Fig 1). After the attractiveness evaluation, the participant was asked the familiarity of the picture. The instruction “If you know the person in the picture or you have seen the picture, check YES. Otherwise check NO.” (in Korean) was given before the task; then, during the task, for each trial the question “Did you know the person in the picture?” was displayed on the screen with yes/no choices. The participant could check yes/no by using the left/right arrow keys. After a trial finished, there was a 1~3 second inter-trial interval (ITI). A fixation cross was presented during the ITI.

The task was performed two times with a week interval on average. For the second day, the task only asked for the attractiveness, since familiarity was already checked on the first day. Upon completion of the second-day task, each picture had two preference ratings for each participant (attractiveness in 1~9 range) and the familiarity answer (whether the subject already knows the person or has seen the picture). To avoid biasing results based on familiarity, familiar pictures were excluded. The two preference-rating scores were averaged for the representative attractiveness of each picture. The average rating score could be a number from 1~9 with 0.5 interval (1, 1.5, 2, 2.5 … 8.5, 9).

The Picture-Rating Task generated a set of pictures with corresponding ratings, whose size was smaller than the original 500. The pictures were sorted based on their rating scores and were split into seven partitions by examining each participant’s ratings distribution and finding natural breakpoints in their ratings across the 1~9 range. The seven partitions were labeled as “1 star”, “1.5 star” … “3.5 star”, “4 star”. Only four groups ‘1, 2, 3, and 4 star’ were used in the subsequent experiment, with the remaining three groups ‘1.5, 2.5, 3.5 star’ excluded to provide a large enough difference of attractiveness levels across the four picture groups.

### Serial-choice task

**Fig 2:**
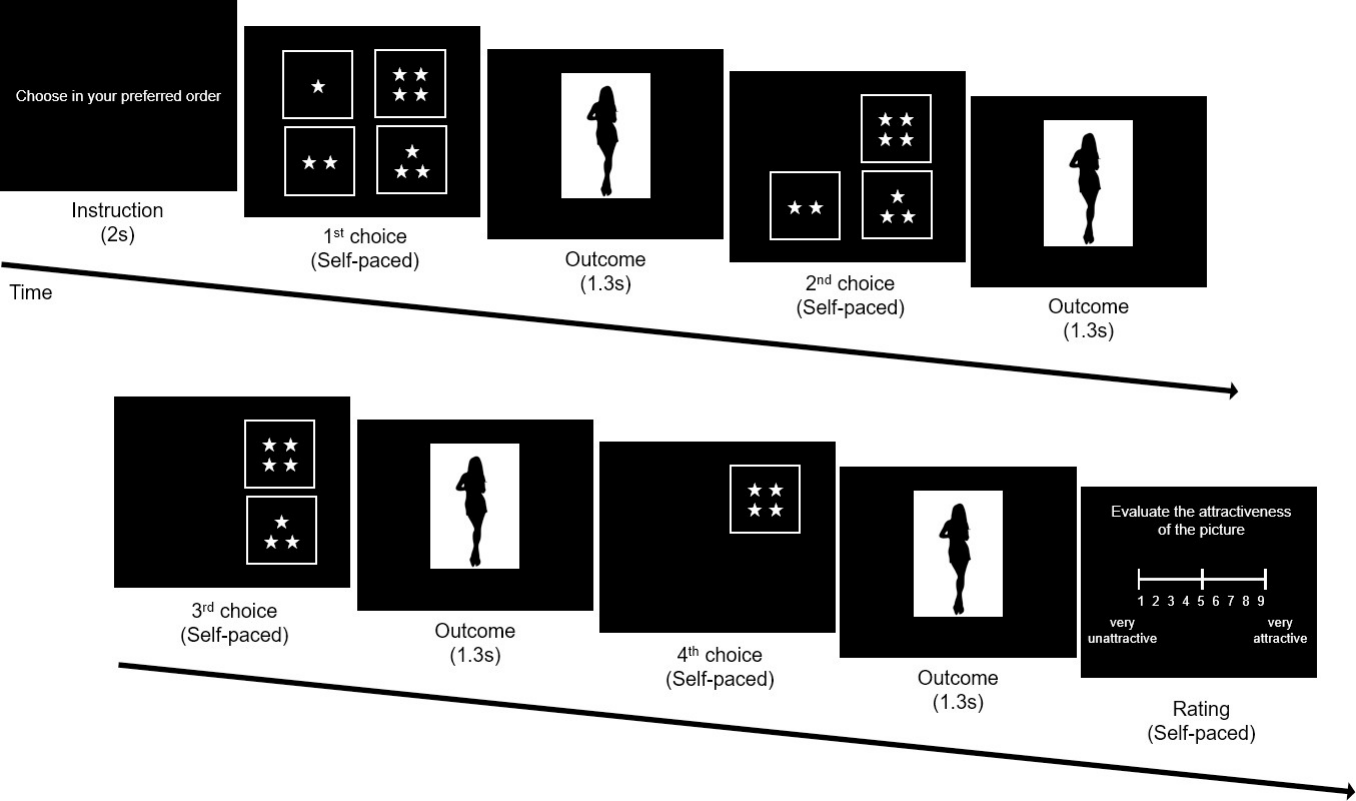
Illustration of the serial-choice task procedure.

For the Serial-Choice Task, the instruction “Choose in your preferred order.” (in Korean) was first presented on the center of the screen for 2 seconds. Then the four choice options appeared, positioned up-left, up-right, down-left, down-right (Fig 2). Each choice option representing the four picture groups from “1 star” (lowest attractiveness) to “4 star” (highest attractiveness) was displayed by the number of “☆” symbols inside a square outline (see Fig 2). The position of each option varied randomly on each trial. The participants were instructed to use four keys on the keyboard (i.e., “r”, “y”, “c”, “b”), with the position of the keys matched with the options on the screen. When the participants pressed one of these keys, a picture from the corresponding ‘star’ group was randomly selected and presented immediately in the center of the screen for 1.3 seconds. Afterwards, the screen turned back to the choice phase, with the selected option removed and only the other options remaining. After the participants selected the final (4^th^) option, the instruction “Evaluate your overall satisfaction of these 4 pictures.” (in Korean) appeared on the screen with the same scale bar used in the Picture-Rating Task (Fig 2). There was no time limit during the task. A total of 20 trials was conducted.

From the order of choices of each participant, we calculated an *SC score*, which was the slope of the linearly best-fitted line of the four data points, with each data point representing the order of choice (x-axis) and average number of stars selected, based on 20 trials (y-axis). For example, if a participant chose the order of “1 star” → “2 star” → “3 star” → “4 star” every trial, the SC score value was +1. In the opposite case, the value was -1. Therefore, the SC score was a number between -1 ~ +1. The more positive value implies the greater tendency to choose the best option last (favorite-last), and vice versa for favorite-first. To test the selection tendency of serial-choice behavior statistically, the empirical data (slope, i.e., SC score) was compared to the average slope from a matched number (69 participants x 20 trials = 1,380) of random (4-length) choice sequences using the Mann-Whitney U test.

To examine individual differences in choice strategy, we divided the participants into seven groups along the SC score. The number of groups (7) was chosen based on the number of the participants whose SC score was negative (thus, based on a natural partitioning of the data). A non-parametric Kruskal-Wallis H test was conducted with a post hoc Mann-Whitney U test comparison with Bonferroni correction to compare the effect of serial-choice strategy on reaction times.

### Sequence-rating and working-memory tasks

**Fig 3:**
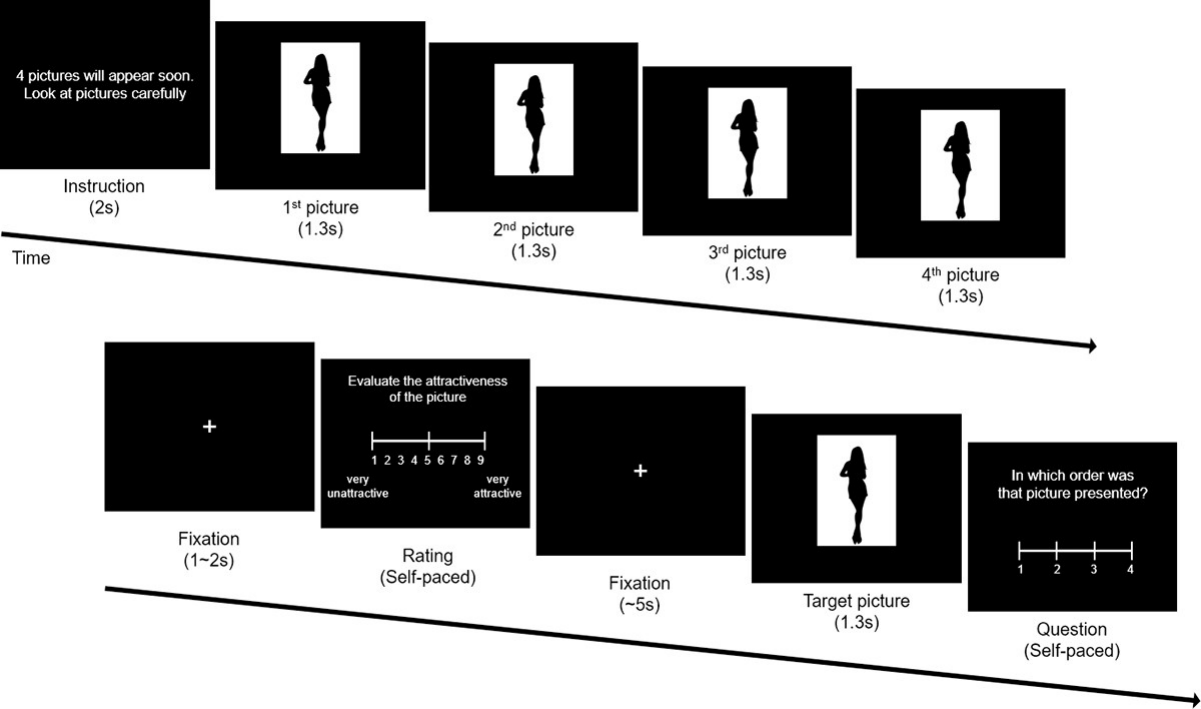
Illustration of the sequence-rating task procedure.

To examine the potential effects of the “peak-end rule”—in which the most salient (peak) and most recent (end) events have greater impact on our overall evaluation of a sequence of experiences—we designed the Sequence-Rating Task that measured the participants’ retrospective evaluation of four sequentially displayed pictures. During the task, the instruction of “4 pictures will appear soon.” (in Korean) was displayed. “Look at the pictures carefully.” (in Korean) was then displayed on the screen for 2 seconds, and then four pictures were sequentially presented for 1.3 seconds each. There was no blank or break between the pictures. After the last (4th) picture disappeared, the query “Evaluate your overall satisfaction of these 4 pictures.” (in Korean) was displayed with the same scale bar used in the Picture-Rating Task. The means of selecting and confirming a number was identical to the previous task. After the participant’s rating was obtained, a fixation cross was displayed for a variable duration of less than 5 seconds. After the rating phase, a target picture, which was pseudorandomly selected from the previous four pictures, appeared. This phase was included to measure the participants’ working-memory performance, and thus constituted the Working-Memory Task. After a 1.3 seconds presentation of the target picture, the question “In which order was that picture presented?” (in Korean) appeared. This second question appeared at least 5 seconds after the previous four pictures were shown, to prevent the participant from not answering the first question carefully, and to produce a higher level of difficulty for the second (working-memory capacity) question. The participants were asked to use the 1~4 number keys in the same way as the previous question. The ‘target picture’ was selected evenly from each of the four positions in the sequence, though this fact was not noticeable to the participants. There was no time limit during the task. The second question was imposed so that participants would focus more intently on the four pictures, as well as to measure working-memory performance (Fig 3).

As mentioned above, the Serial-Choice (20 trials) and Sequence-Rating (96 trials) trials were interleaved within the same session. This intermixed Serial-Choice / Sequence-Rating Tasks had a total of 10 sessions. Each session was composed of 11~12 trials, took approximately 5 minutes to complete, with 1-minute breaks between each session. The ITI varied between 2~5 s. A fixation cross was presented during the ITI.

In the Sequence-Rating Task, we tested sequences with a wide range of slopes: mean (M) = 0.021, standard deviation (SD) = 0.856, range: -2.85~+2.70. (Note that for greater precision, the slopes were calculated based on the actual rating scores of the pictures from the Picture-Rating Task, not by the number of stars of their corresponding category). The total number of trials for the Sequence-Rating task was 96. In addition, to provide a cleaner measure of the actual overall sequence rating, the difference between the rating score of the overall sequence itself and the average rating score of the four pictures of that sequence (based on the Picture-Rating Task results) was calculated and used as the dependent measure. For example, if a sequence of four pictures contained pictures with ratings 2.5, 4.5, 6.5, and 8.5 individually (note that these are hypothetical but possible ratings of the pictures per se, not the subsequent ‘star’ category to which each belonged), its mean rating score would be 5.5. If a participant rated this sequence as 7, the difference is +1.5 (7–5.5). Note that this difference could be negative with an overall sequence rating less than 5.5. To examine and analyze the results, first, for each participant we plotted these difference values (as the dependent measure) against the slope (from negative to positive) of each tested sequence and calculated the best-fit line, with the slope of this line revealing the participant’s general preference for ascending (increasing, with positive slope) or descending (decreasing, with negative slope) sequential experiences. We called this overall slope of the best-fit line the *SR score*, the value to capture each participant’s sequence-rating preference. At the population level, we also compared the empirical data (i.e., average of all SR scores) with a randomly generated pair (69 participants ⅹ 96 trials = 6,624 rating scores) to examine whether the overall sequence preference was significantly different form random behavior (using an independent samples t-test). Given the comparable measures and analyses between sequence rating (SR score) and serial choice (SC score), it is important to distinguish between them, with sequence rating reflecting retrospective (and thus passive) memory-based preferences, and serial choice reflecting (active) prospective selections [1, 13].

For the Working-Memory Task, the average percent correct (i.e., accuracy) of the second (working memory) question was calculated for each of the four positions of the target picture in the previously presented sequence (1st, 2nd, 3rd, and 4th). We applied the non-parametric Friedman test followed by a post hoc Wilcoxon test with Bonferroni correction to compare both the rating score and the working-memory test results.

### Delay-discounting task

**Fig 4:**
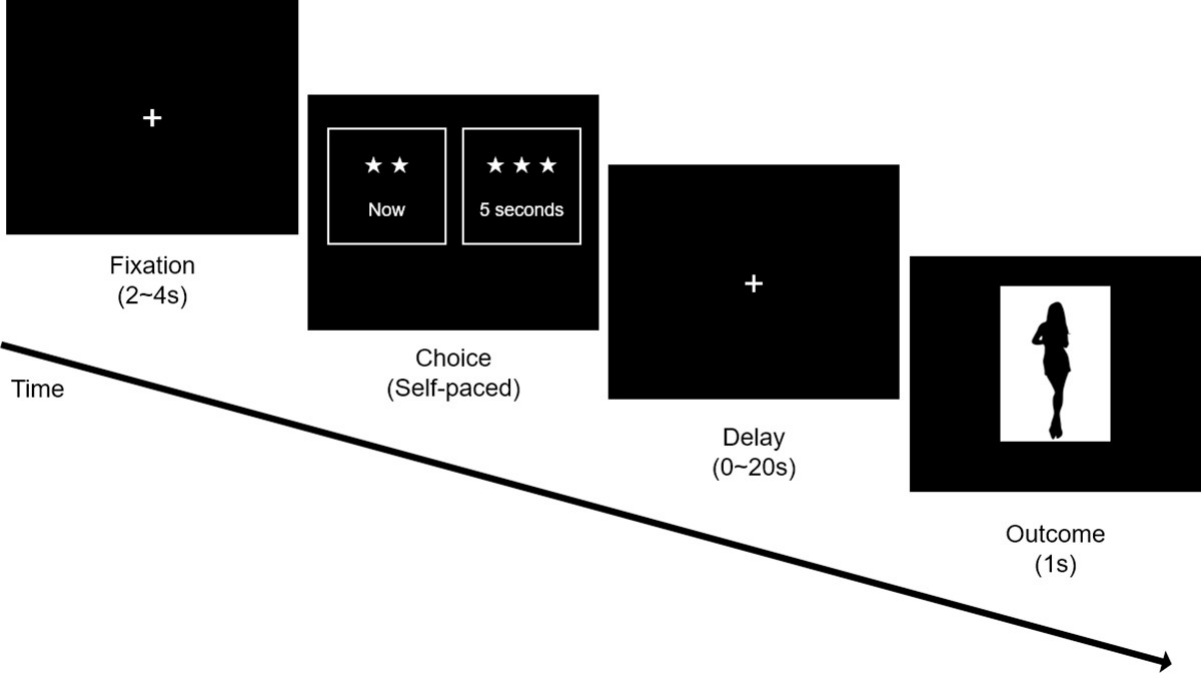
Illustration of the delay-discounting task procedure.

To test if serial choice can be explained by the same underlying mechanism as delay discounting, it required both a realistic task that would clearly evoke the delay-discounting phenomenon, as well as one that could directly test both delay discounting and serial choice. Thus, the Delay-Discounting Task required immediately consumable rewards and real-time delay to obtain the reward, leading us to develop the Picture-Rating Task. The picture set and its classification criteria were the same as the previous tasks. During the task, two square-shaped targets appeared horizontally on the left and right side of the screen (Fig 4). Each target contained the information about the amount of delay and level of attractiveness of the picture offered for viewing on that trial. One of the two targets offered a 1-second presentation of a ‘2 (or 3) star’ picture immediately (smaller/sooner, SS); the other target offered a presentation of a ‘3 (or 4) star’ picture with the same duration (1 s) but variable delay (larger/later, LL). There were three conditions for relative attractiveness between pictures in the experiment: ‘2 vs 3 star’, ‘3 vs 4 star’, ‘2 vs 4 star’. We excluded the ‘1 star’ picture set to simplify the experiment and minimize overall session duration. The amount of delay for the LL options varied thus: 1, 3, 6, 9, 11, and 20 seconds—with each delay tested five times, in five sequential sessions. That is, there were three reward conditions and six delay variable values, producing 18 trial types, presented together pseudorandomly in five sequential sessions, with a 1-minute break between each session. In addition, we also included five ‘catch trials’, one per session, which offered a larger (i.e., more attractive) option with no time delay (i.e., 2 star immediately vs. 4 star immediately), which thus necessitated the participants to choose the LL option. Thus, a total of 95 trials were conducted across five sessions.

Participants were required to choose one of the targets with no time limit. The locations of the targets (left, right) varied randomly on each trial. Selection of either target was followed by presentation of a picture after the offered delay time. After the presentation of the picture, the ITI was followed with a random duration between 2~4 seconds. A fixation cross was shown during the ITI.

As typical, to capture the degree of discounting in each condition (e.g., 2 vs 3 star), we used an indifference-point procedure, which provides a measure that represents the length of the larger reward delay at which preference for the SS and LL options was 50/50. First, we obtained the discounting curve by plotting each participant’s average proportion of SS vs. LL choice (y-axis) for each of the six delay values within each condition (x-axis). Then, hyperbolic discount parameters (Mazur’s 1987, k-value) were calculated for each participant in each attractiveness condition. The discount function was assumed to be the equation: v' = v / (1 + kD), where v’ indicates discounted utility of the picture; v indicates picture utility (assumed to depend linearly on the number of stars); k is the discount parameter; and D is the delay length (1~20 s). We also calculated the area under the curve (AUC) [28], which is an atheoretical index for quantifying the extent of an individual’s propensity for discounting. AUC estimates the range from 0 to 1, and differently from the k-value, small numbers indicate more impulsive choice (the SS option) in delay discounting. Since these two alternative analytic methods yielded similar results, we only report the k-value here—i.e., the discounting parameter for each of the three conditions averaged across participants—with a larger k-value signifying greater discounting.

Since the purpose of the catch trials was to ensure that our participants engaged in the task with proper motivation and attention, we excluded the data from participants who chose a disadvantageous option more than once in a catch trial. One participant was excluded from further analysis. We also used an additional criterion to screen for inappropriate responses in the study (e.g., due to lack of motivation). Since it is natural to assume that one’s subjective value decreases with longer delays, each indifference point is expected to be lower than the preceding ones. Therefore, if we found an indifference point 20% higher than the preceding point more than once, the participant was removed from the study. However, no participants violated this criterion. Finally, we applied a non-parametric Friedman test followed by a post hoc Wilcoxon test with Bonferroni correction to compare the k-value and reaction time of each condition statistically.

### Effort task

To validate the rewarding effects of the picture stimuli, as well as to verify whether our participants maintained sufficient motivation during our study, we conducted Effort Task sessions between the Serial-Choice and Sequence-Rating Task sessions in the 3rd day of the experiment. The basic process of the task was similar to that in a previous study [19]. In our Effort Task, pictures were selected evenly from four attractiveness conditions (1 star ~ 4 star), which again was not noticeable to the participants. On each trial, a picture was shown for 0.8 seconds followed by a 10-second blank period. By pressing the space key and backspace key in sequence, participants could make the same picture reappear for another 0.5 seconds. To make the task more difficult, participants were instructed to use only one finger. Participants could press the buttons as many times as they wanted, within a 10-second duration time limit. Each session consisted of 12 trials with no break, and participants performed a total of 3 sessions. The average number of times the participants pressed the two-button-sequence (to view the picture again for 0.5s within the 10-second trial duration) was calculated for each star category. We applied the non-parametric Friedman test followed by a Wilcoxon test with Bonferroni correction to compare the average number of (two-button-sequence) responses for each category statistically.

### Questionnaires

On the 1st, 2nd, and 4th days of the experiment, between and after the above mentioned task sessions, the participants worked on and eventually completed the following four questionnaires: (1) the Big Five Inventory (BFI) [29, 30], (2) the Emotion Regulation Questionnaire (ERQ) [31, 32], (3) the Beck Depression Inventory II (BDI II) [33, 34], and (4) the State-Trait Anxiety Inventory (STAI) [35, 36]. Basic demographic information (age, education years, and BMI) was also collected on the 1st day of the experiment. Scores on all of these scales are summarized in S2 Table (Measures 8–12).

## Results

### Picture-rating task

Since all of our experimental tasks relied on the picture stimuli as reward, validation of the Picture-Rating Task results was essential. The mean rating score (from the 1~9 available range) of the entire picture set (N = 500) was 5.29 (SD = 2.17), while the mean reaction time was 2.54 seconds (SD = 0.95). There was a significant correlation between rating scores on the 1st and 2nd days (Pearson-r = .746, p = .000), indicating that the participants exhibited consistent, reliable preferences for the pictures comprising the picture set.

To eliminate effects of familiarity, we removed any images of the women identified by participants. The mean number of familiar pictures across participants was 18.42 (SD = 22.48) (thus, on average, each participant recognized the woman in ~18 pictures). As described, we then split the pictures into seven partitions based on their ratings, with S1 Table showing the mean number of pictures for each “star” group. From this, we used only the 1, 2, 3, and 4 star group pictures further, to provide a clear distinction of attractiveness levels. Once all familiar pictures were removed, the average number of pictures for the 1, 2, 3, and 4 star groups was above 50 (S1 Table), which provided a sufficient number of pictures to run the experimental procedures.

### Effort task

**Fig 5:**
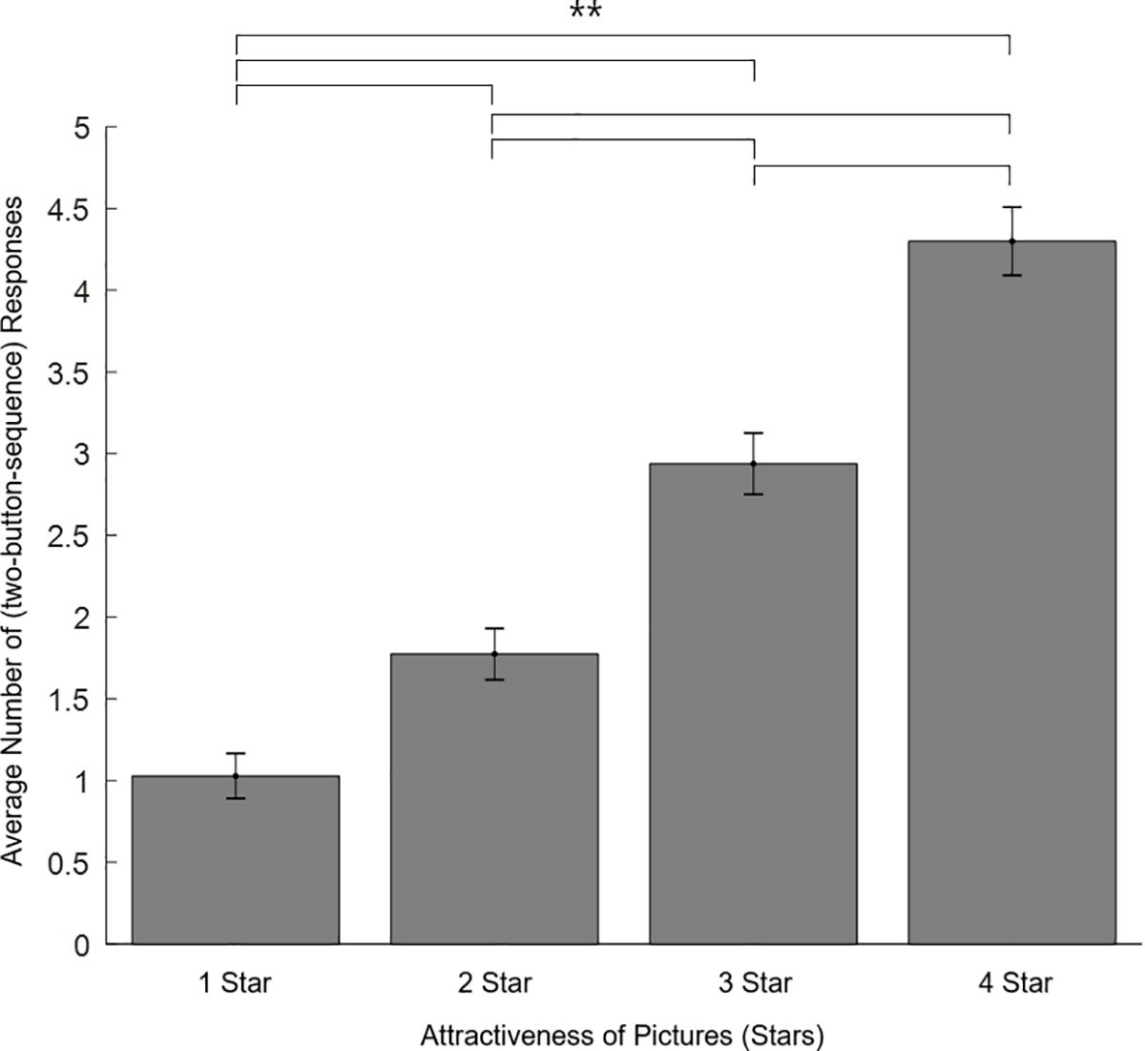
Average number of (two-button-sequence) responses in the Effort Task (i.e., the average number of times the participants pressed the two-button-sequence to view the picture again for 0.5s within the 10-second trial duration) for each category. The effect of attractiveness level was found to be significant, indicating that the classification process for picture sets worked reliably, providing a clear distinction between each category, and providing evidence that the pictures were treated as consummatory rewards (to be worked for). The error bar denotes standard error of the mean. (** p < .000 for all pairs).

For further validation of the picture stimuli—beyond the reliability of the images over repetition and time, with two Picture-Rating-Task sessions required, and an average of one week between days—we developed the Effort Task, which determined whether the picture stimuli actually evoked a rewarding effect that accorded with the ‘star’ classifications. Fig 5 shows the average number of (two-button-sequence) responses in the Effort Task for each condition. Indeed, the number of responses increased with the level of attractiveness of the picture groups: “1 star”: 1.03 ± 1.23 (mean ± standard deviation), “2 star”: 1.77 ± 1.40, “3 star”: 2.94 ± 1.69, “4 star”: 4.30 ± 1.86. Non-parametric Friedman test of differences among repeated measures was conducted and rendered a Chi-square value of 183.823, which was significant (p = .000). Post hoc Bonferroni Wilcoxon signed rank test showed the difference in the number of responses was significant between each condition pair (i.e., each star category) (p = .000 for all pairs). In addition, there was a significant positive correlation between number of responses and stars in each condition (S2 Table).

A closer examination of the results also provides a clearer sense of the degree of effort exerted. In the task, participants were required to make a two-button-sequence response (‘space’ then ‘backspace’ keys) to view the picture again for 0.5s as many times as desired, within the 10s trial duration. The 4.29 response (i.e., 8.58 button presses) average in the 10s duration for the 4-star category indicates that the participants continued to press the two-button sequence within 2s throughout the trial (with the time of picture viewing 2.15s on average). Given that the participants chose to continue the two-button-sequence response to view the pictures rather than do nothing and rest, again demonstrates the motivational incentive of the images. Overall, then, the results thus show that the opportunity to view the opposite-sex pictures served as an incentive for effort, which in turn indicates that the picture stimuli indeed had a rewarding effect, with the difference between the number of stars properly reflecting different levels of reward.

### Serial-choice task

Since our Serial-Choice Task collected the participant’s behavioral data repeatedly (20 trials), we first examined if learning may have changed preferences during the task. However, the slope of the first trial (i.e., number of stars of choice across the choice sequence: 0.473) and the average across all trials (0.432) were not significantly different (Wilcoxon: Z = -1.565, p = .118), and were highly correlated (Pearson-r = .679, N = 69, p = .000). To look in finer detail across the entire session, we calculated the slope of each trial (again, the number of stars of choice across that trial’s choice sequence), for all 20 trials of the session, and then found the best-fit line to these 20 slopes plotted across the session. There was no significant difference (Mann-Whitney: U = 3115.50, p = 773) between the mean slope value (M = .004, SD = .029) and its random pair—i.e., 1,380 random (4-length) choice sequences, divided into 20 groups (with average value of each group representing a trial), then calculating the slope of the best-fit line—(M = .006, SD = .015). Therefore, we found no evidence for learning effects across the session, with the results thus well-capturing a stable sequence preference.

**Fig 6:**
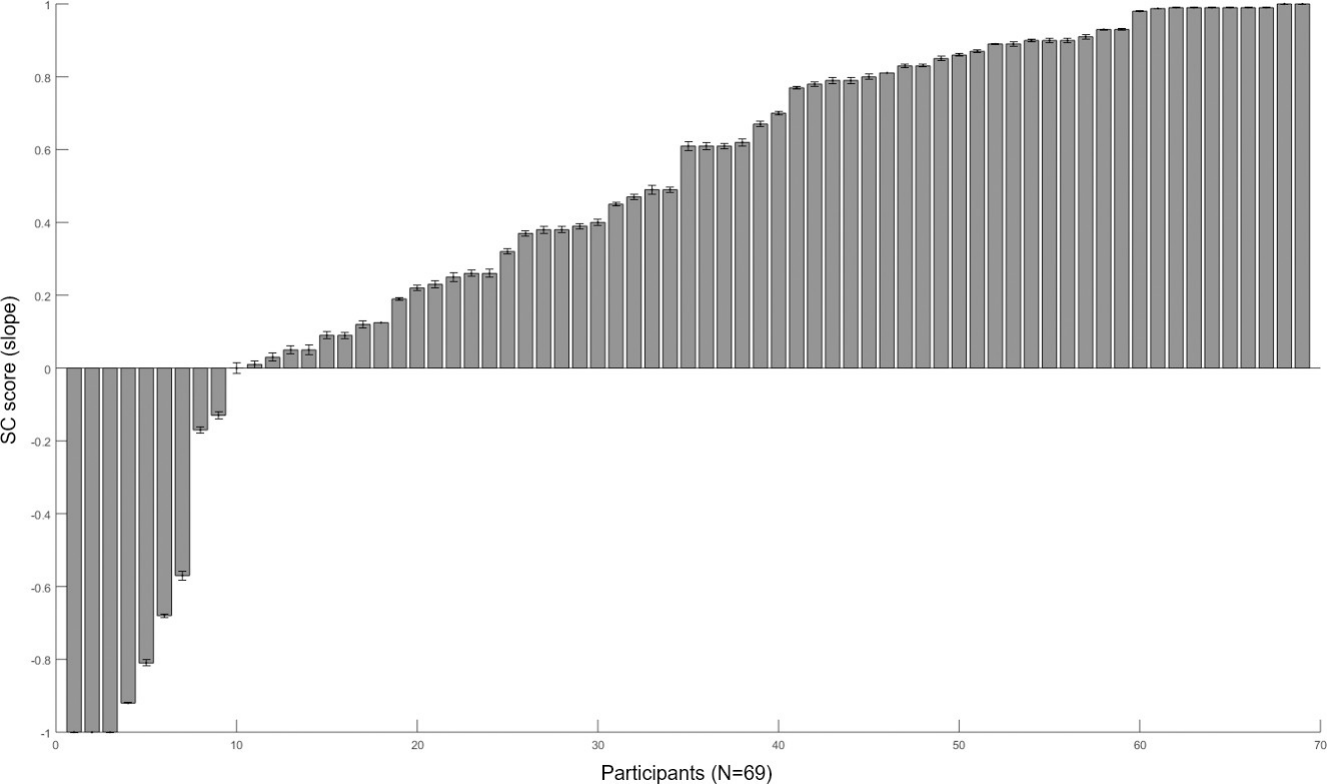
Individual results of the Serial-Choice (SC) task. The vertical axis represents the SC score, which is the slope of the best-fit line of the participant’s average choice of stars for each order position within the sequence. The horizontal axis represents each participant, aligned by the SC score. Higher (close to +1) SC scores represent the tendency toward favorite-last (i.e., 1-2-3-4) preference, while the opposite (close to -1) represent favorite-first (i.e., 4-3-2-1) behavior. The error bar is standard error of the mean. Favorite-last was the dominant strategy, while a distinct proportion of favorite-first behavior also existed.

To measure the serial-choice behavior, we calculated the SC score. The SC score (vertical axis in Fig 6) represents the slope of the best-fit line of each participant’s average choice of stars for each order of choices in the Serial-Choice task. A larger (close to +1) SC score means tendency toward favorite-last (1-2-3-4) behavior. A smaller (close to -1) score means the opposite: toward favorite-first. Fig 6 shows the results of the Serial-Choice task, with each participant represented by a single bar. The tendency toward favorite-last behavior was dominant among the participants, while favorite-first behavior also existed in a distinct population.

**Fig 7:**
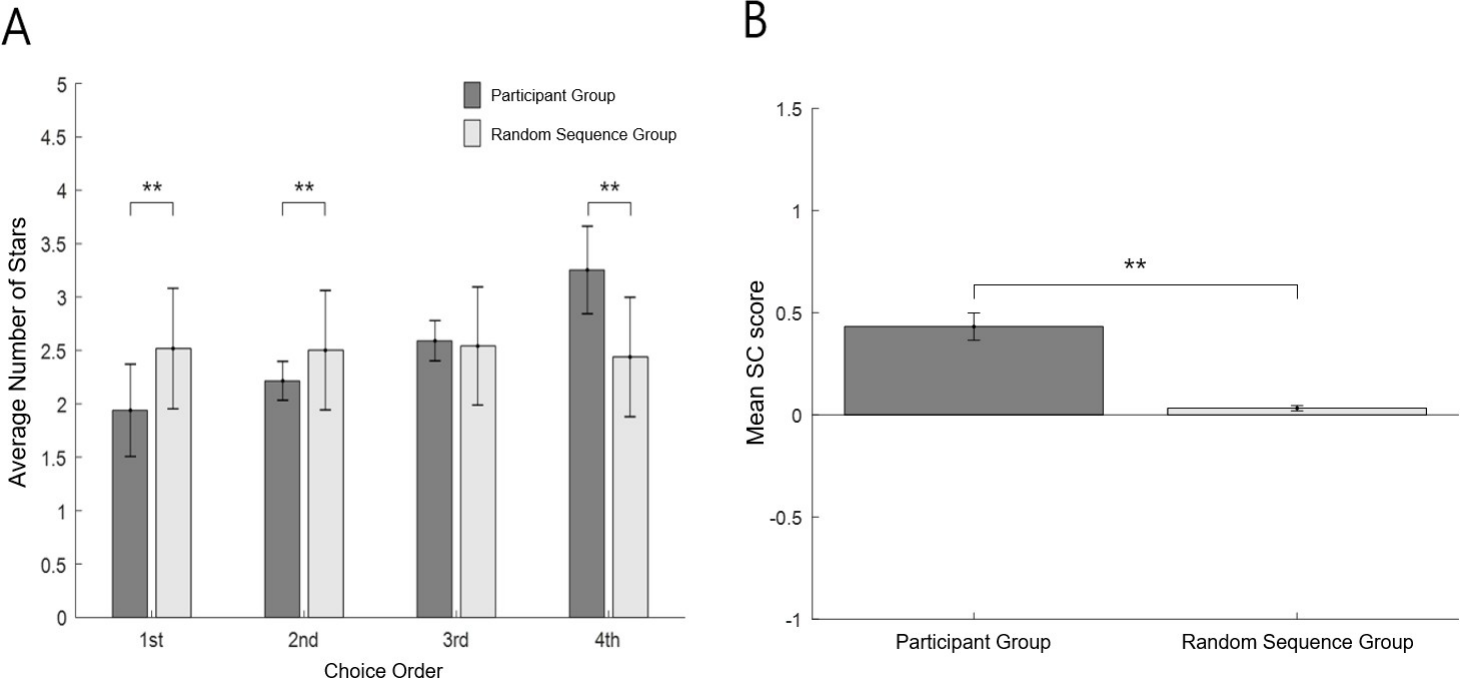
Comparison of SC task results with a random sequence group. The difference between the empirical SC Task data and its random pair (i.e., the average of 1,380 randomly generated four-length sequences). Panel A shows the average number of stars for each order position within the sequence. The average number of stars for 1^st^ choice was significantly lower and for 4^th^ significantly higher than its random pair (p = .000). Panel B shows the mean SC score of the participant group and its random pair. The slope from our empirical data was significantly higher than the slope of random sequences (p = .000). The error bar is the standard deviation. These results indicate that the favorite-last choice was the dominant strategy for our participants. (** p = .000).

To test whether the serial-choice results reflected a meaningful pattern versus random behavior, we compared the empirical data with randomly generated sequences. Fig 7A shows the average number of stars in the Serial-Choice task for each choice order, both derived from our participants’ behavior (left) and randomly generated sequences (right). The average number of stars in the 1st choice was significantly lower than its random pair (Mann-Whitney; U = 1215.50, p = .000), while the 4th choice was significantly higher (U = 868.50, p = .000). The 2nd choice was also significantly lower than its random pair (U = 1415.00, p = .000). Fig 7B shows the overall mean SC score (i.e., slope derived from Fig 7A results) of our participant group and its random pair. The slope from the empirical data was significantly higher than the slope for the random sequences (p = .000). This again indicates that the favorite-last choice was the dominant strategy of the participants, being significantly different from randomly generated results.

Besides SC score, we also calculated the standard deviation (SD) of the slope to determine how strict the participants’ serial-choice strategies were—i.e., the consistency of their preferences across trials. Hence, the higher SD value (vertical axis) means more varied strategies were used across the 20 trials of the SC task. More specifically, for example, if one participant maintained the same strategy (e.g., ‘4-3-2-1’ favorite-first *or* ‘1-2-3-4’ favorite-last) every trial, the SC score would be low (-1) or high (+1), respectively, while the SC standard deviation (SD) would be low (0) for both. In contrast, if a participant followed, say, both extreme strategies evenly (1-2-3-4 for half the trials, and 4-3-2-1 for the other half), the SC score would be close to zero, while the SC SD would be high (1.026). S1 Fig shows the results of the SC SD, which were aligned using the same order obtained from Fig 6 —thus, each single bar represents the same participant in Fig 6. Since the favorite-last strategy was dominant in our population (thus with high SC), there was a significant negative correlation between the SC slope and the SC SD (Pearson-r = -0.238, N = 69, p = 0.049): thus, those with a high SC exhibiting a low SD, and thus tending to maintain the same strategy throughout. Nonetheless, as described, the two extreme strategies (favorite-fist and favorite-last) that were both prominent in the results, by definition, resulted in lower SDs, producing two general classes of stable behavior: an extreme, *singular* strategy versus *mixed* ones.

**Fig 8:**
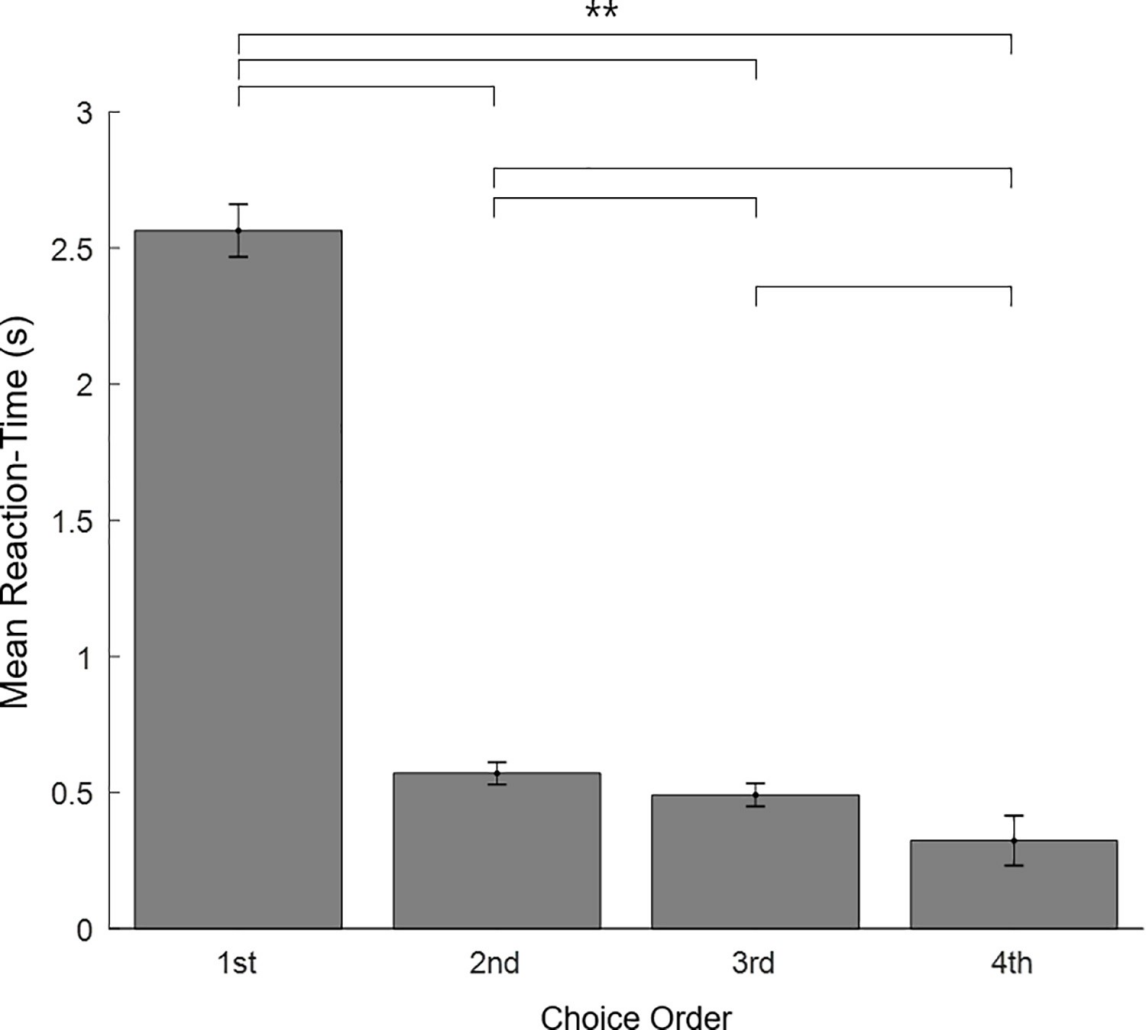
Reaction times of serial-choice task. The horizontal axis represents the order of choices and the vertical axis is mean reaction time. The reaction time of the first choice (M = 2.564) was significantly longer than the others (p = .000). This suggests that the participants planned the order of choices before their first choice. The error bar denotes standard error of the mean. (** p = .000).

To further characterize the serial-choice behaviors of the participants, we measured the reaction time during the SC task. In Fig 8, the horizontal axis represents the order of choices and the vertical axis represents reaction time. A non-parametric Friedman test of differences among repeated measures was conducted and rendered a Chi-square value of 175.07, which was significant (p = .000). While the reaction time was significantly decreased as the order of choice progressed, the reaction time of the 1st choice (M = 2.56, SD = 1.11) was particularly longer than the others (2^nd^: M = 0.57, SD = 0.32, 3^rd:^ M = 0.49, SD = 0.26, 4^th^: M = 0.32, SD = 0.20). This reaction-time result suggests that the participants were planning the order of the entire choice sequence prior to their first selection; and thus provides some evidence that the entire sequence was viewed as a singular overarching event (as opposed to individual, singular choice events).

**Fig 9:**
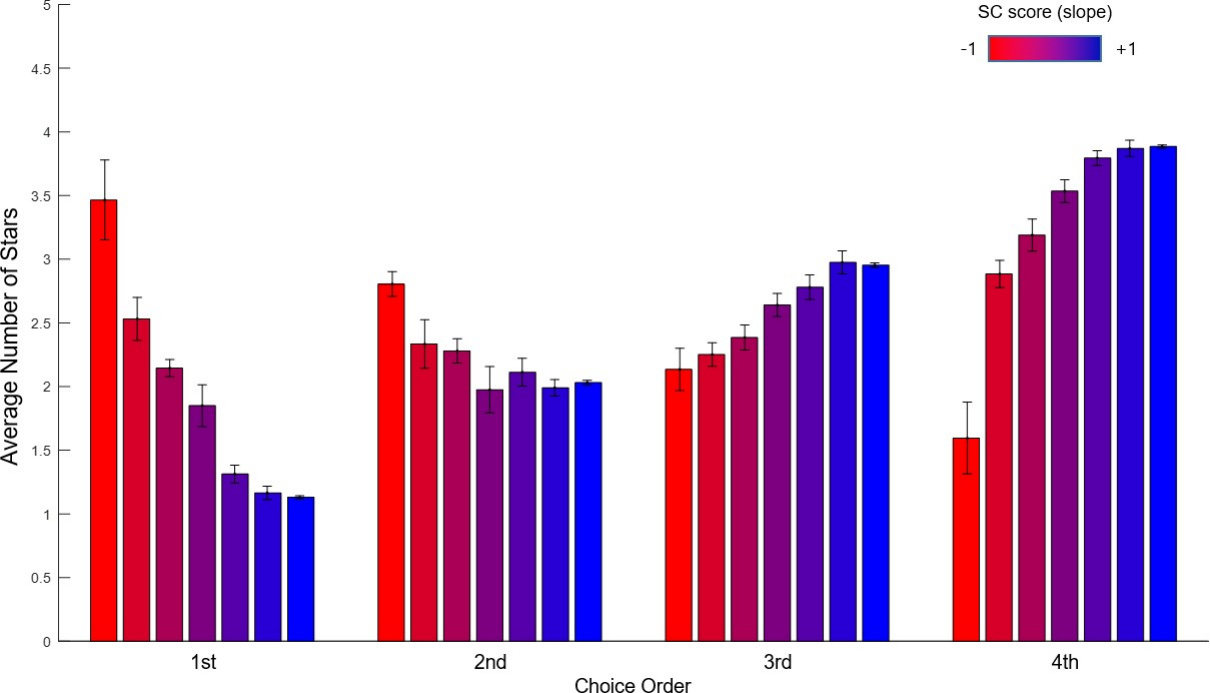
Average number of stars of each choice for different SC scores. The vertical axis represents the average number of stars, while the horizontal axis represents the order of choice in the SC task, with participants divided into seven groups (red to blue) along the SC score (-1 to +1). The first (red, far left for all four order positions) group exhibited strict favorite-first behavior while the other groups behaved oppositely (except the second group). The second group exhibited a unique behavioral pattern, which was U-shaped (e.g., 4-1-2-3), although their SC slope had a positive number since they also mostly picked their best option last. These results indicate that there were clear preferences among the participants for both favorite-first and favorite-last strategies, as well as mixed strategies, although favorite-last prevailed overall. The error bar denotes standard error of the mean.

Although favorite-last was the most dominant strategy found, there was a distinct population of favorite-first preferences as well. To characterize this more clearly, we divided our participants into seven groups along the SC score, based on the number of participants with negative SC scores (N = 9 out of 69). Fig 9 shows the average responses in the SC task for each of the seven groups across choice order. The first (red) group clearly exhibited favorite-first behavior, choosing their most favored option first then choosing in descending order, while the other groups behaved differently, including the last one (blue) behaving in the opposite way (favorite-last). Interestingly, the second group exhibited a unique behavioral pattern, which was U-shaped (e.g., 4-1-2-3), although their SC slope had a positive number since they also mostly picked their best option last. These results indicate that there were clear preferences among the participants for both favorite-first and favorite-last strategies, as well as mixed strategies, although favorite-last prevailed overall (see S2 Fig for more detail).

**Fig 10:**
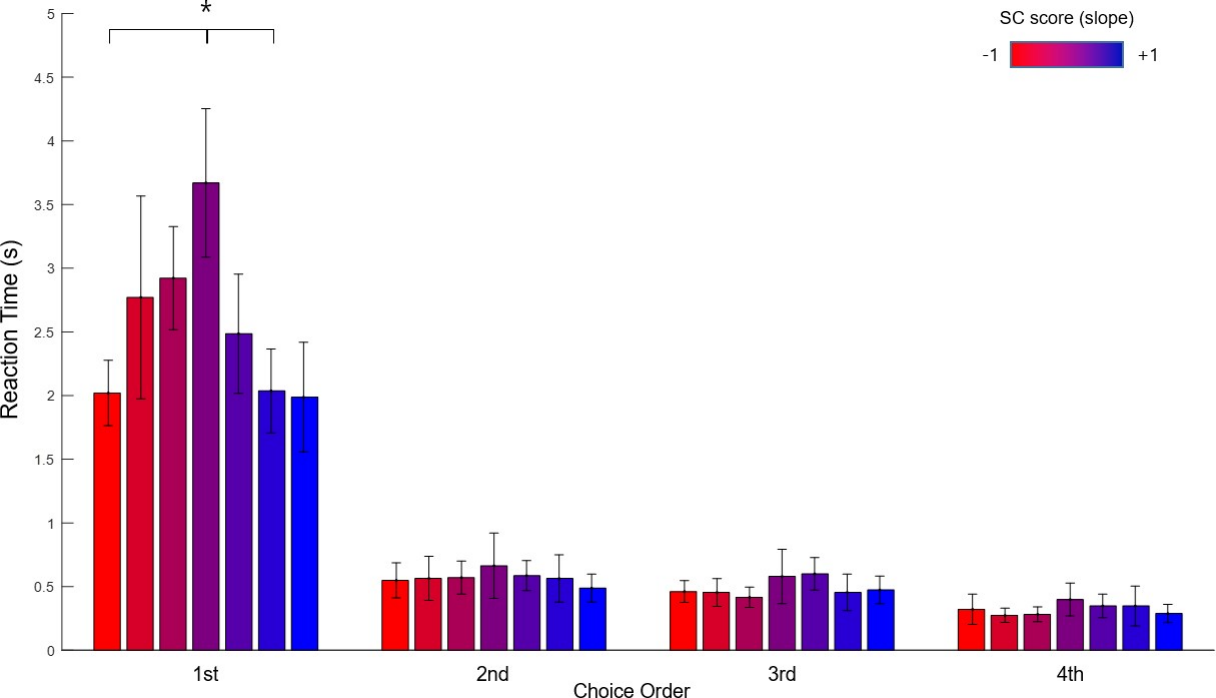
Reaction-time of serial-choice task together with SC score. The vertical axis represents the mean reaction time and the horizontal axis represents the order of choice in the SC task, with participants divided into seven groups (red to blue) along the SC score (-1 to +1). In the first position of the choice sequence, the middle (purple) group exhibited a significantly longer reaction time than the first (far left, red) and the last (far right, blue) groups. This suggests that the middle group in particular (and the other middle groups more generally) took more time to decide the serial-choice strategy. The error bar denotes standard error of the mean. (* p < .002).

We also compared the reaction times for the seven groups, to examine the patterns more closely (Fig 10). Clearly, the reaction times for the first choice were longer than the others for all groups, suggesting that in every case, subjects on average determined the overall response strategy prior to selection—evidence again that the entire sequence was viewed as a singular overarching event. Nonetheless, compared to the first (red) and last (blue) groups, the middle (purple) group exhibited a significantly longer reaction time for the first selection (χ^2^(6) = 23.30, p = .001). Post hoc comparisons using a Mann-Whitney U test with Bonferroni correction showed that the reaction time of the middle (4th) group (M = 3.76, SD = 1.08) was significantly longer than the 1st (M = 1.93, SD = 0.47) (U = 3.00, p = .001), and 6th (M = 1.88, SD = 0.27) (U = 0.00, p = .000) groups. This suggests that, over and above the first and last group, the middle group took significantly more time to consider their overall choice strategy. The difference between the 4th group and 2nd (M = 2.80, SD = 1.59), 3rd (M = 2.85, SD = 0.81), 5th (M = 2.49, SD = 0.93) or 7th (M = 2.17, SD = 0.99) was not significant.

### Sequence-rating task

Because of the obvious similarities of sequential choice and retrospective sequence ratings that lead to the ‘peak-end’ bias effect, to compare the phenomena we also conducted a Sequence-Rating Task, which presented four picture stimuli in sequential order then asked the participants to rate their overall satisfaction with the experience. First, as an internal consistency check of the reliability of the task itself, we examined the correlation among the rating scores of each sequence and the mean rating score of the pictures in the sequence (the latter from the Picture-Rating Task). All participants had a significantly positive correlation (p < .05), except for three. However, since the exclusion of the three participants did not make a difference in the results, we included all participants for further analysis.

**Fig 11:**
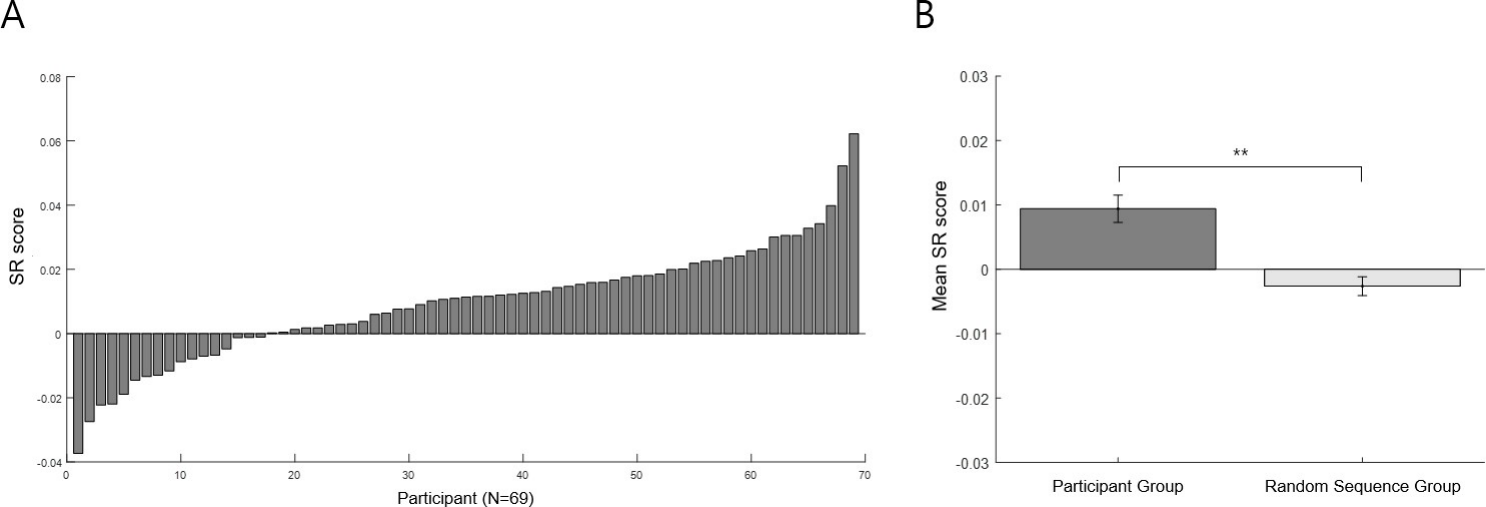
Sequence-Rating task results. Panel A shows the results for each participant, with the vertical axis the SR score (i.e., from the best-fit line of the sequence-rating dependent measure across the tested sequences). The horizontal axis represents each participant, aligned by the SR score (i.e., the same participant order as Fig 6). The higher (positive) SR score reflects a preference for ascending (e.g., 1-2-3-4) sequences, while the opposite (negative) reflects a descending (e.g., 4-3-2-1) preference. Preference for ascending sequences was dominant, while a distinct proportion preferred descending. Panel B shows the mean SR slope of all participants versus its random pair. The empirical data slope was significantly higher than from random sequences (p = .000). The error bar is the standard deviation. Both results show a prevailing preference for favorite items coming later in the sequence (i.e., rather than first), notwithstanding the distinct group who preferred a descending order. (** p = .000).

To obtain a measure of each participant’s general preference for retrospective sequential experiences, we calculated the SR score, which is the slope of the best fitting line of the sequence-rating dependent measure (i.e., the difference between the rating score of the sequence and the average rating score of the four pictures in the sequence from the Picture-Rating Task) across the tested sequences for the participant. As seen in Fig 11A, most participants exhibited a positive SR score, while a distinct proportion of them also exhibited a negative SR score. The more prevalent preference for ascending sequences generally corroborates previous findings that people prefer experiences that end well (peak-end) [14, 15]; and at the same time, there exists a distinct population who prefer sequences that begin well—i.e., with a peak at the beginning. It is interesting to note the resemblance between the distributions of the SR (Fig 11A) and SC (Fig 6) scores, with both results sharing the prevalent preference for ascending sequences, with also a population who prefer descending. Whether this resemblance reflected actual individual preferences that remained consistent across the two tasks was tested below (under “Correlation between Serial-Choice behavior and other measures”) (and in fact was not found—see below).

Finally, to test whether the sequence-rating results reflected a meaningful pattern versus random behavior, we compared the empirical data with randomly generated sequences. The average SR score (M = .009, SD = .017) for the entire participant population was significantly different from its random pair (t(68) = -4.25, p = .000) (Fig 11B), and also yielded a significant difference from zero (t(68) = 4.25, p = .000). Thus, there was indeed a general preference for ascending sequential experiences, notwithstanding the smaller group who preferred the descending sequential experiences.

### Working-memory performance

**Fig 12:**
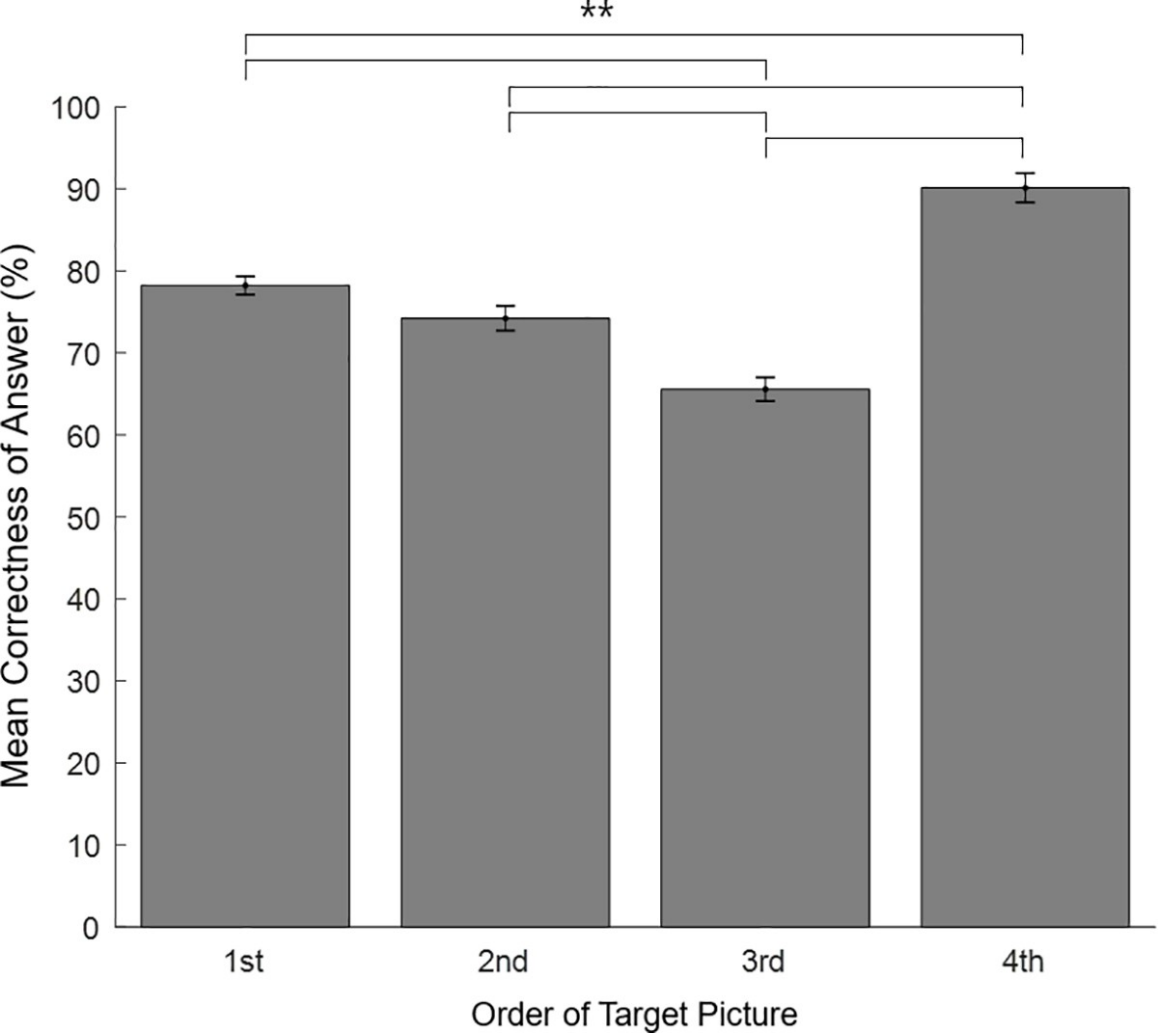
Working-memory performance for order of target picture in the sequence-rating task. Working-memory performance was significantly higher when the target picture was located in the last position of a sequence (M = 0.90, SD = 0.10) than the 1st (M = 0.78, SD = 0.13), 2nd (M = 0.74, SD = 0.13), or 3rd (M = 0.67, SD = 0.13) positions. These results indicate both “recency” and “primacy” memory biases occurred with the sequential paradigm. The error bar denotes standard error of the mean. (** p = .000 for all pairs).

Working-memory performance was highest when the target picture was located in the last position (M = .90, SD = .10) of the sequence, compared to the 1st (M = .78, SD = .13), 2nd (M = .74, SD = .13), and 3rd (M = .67, SD = .13) positions (Fig 12). A non-parametric Friedman test of differences among repeated measures was conducted and rendered a Chi-square value of 101.65 that was significant (p = .000). A post hoc Bonferroni Wilcoxon signed rank test also found the differences to be significant, except between the 1st and 2nd conditions (Z = -2.36, p = .018). The highest answer accuracy of the last position shows a memory “recency” effect, while the lowest accuracy of the 3^rd^ position (than 1^st^ and 2^nd^ position) also reveals a “primacy” effect. These results indicate that both memory biases (primacy and recency) occurred in our sequential experimental paradigm.

### Delay-discounting task

**Fig 13:**
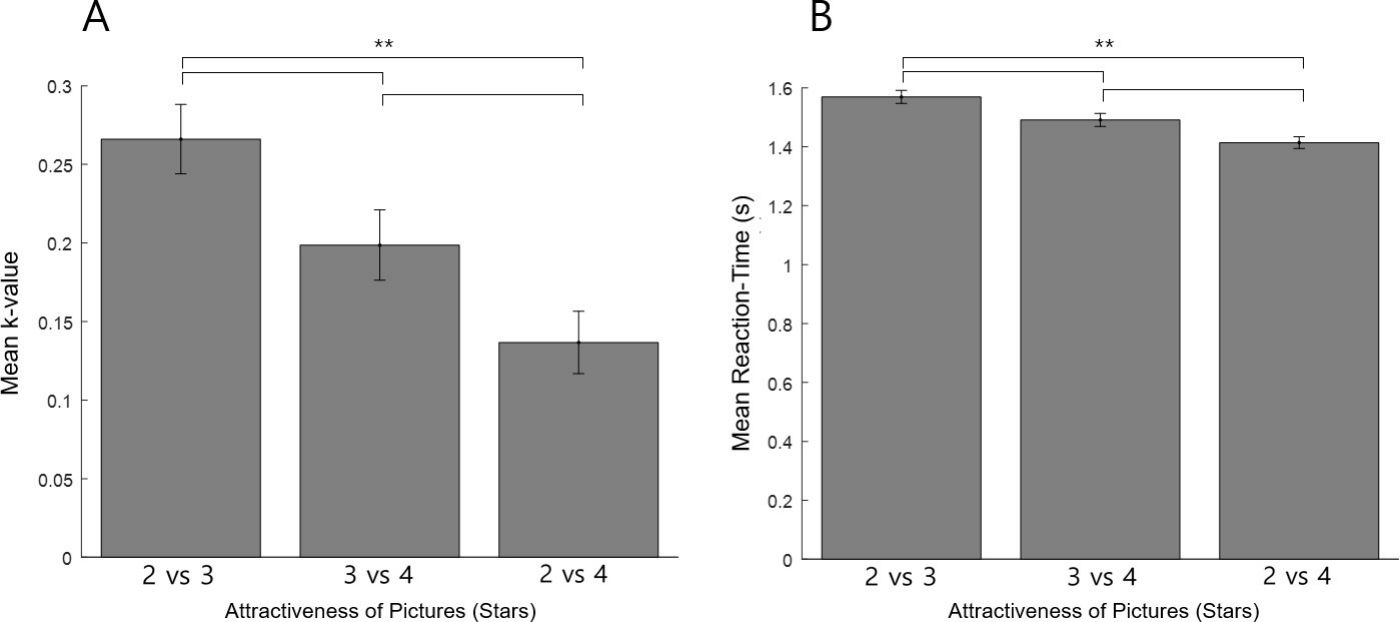
Delay-Discounting task results. Panel A shows the mean k-value (delay-discounting parameter) for each attractiveness condition, with a larger k-value signifying greater discounting. Panel B shows the mean reaction time for each condition. The discounting parameter and reaction time both decreased when the amount of (delayed) reward was larger in both absolute (“2 vs 3” > “3 vs 4”) and relative (“2 vs 3” > “2 vs 4”) terms. These results indicate that participants discounted the delayed, higher-rated pictures properly, and were more willing to wait for the larger reward when it was more attractive. The results verify that the participants actually considered the picture stimuli rewarding according to the “star” categories, validating this critical component of the paradigm. The error bar denotes standard error of the mean. (** p = .000 for all pairs).

Because sequential choice may share similar mechanisms to those that underpin delay-discounting behavior, we also conducted a Delay-Discounting Task with the same picture stimuli. Fig 13A shows the average k-value of our participants for each attractiveness condition (with a larger k-value signifying greater discounting). The k-value was highest for the “2 vs 3” condition (M = .27, SD = .20), lowest for the “2 vs 4” condition (M = .14, SD = .18), and moderate for the “3 vs 4” condition (M = .20, SD = .20). A non-parametric Friedman test of differences among repeated measures was conducted and rendered a Chi-square value of 75.22 that was significant (N = 65, p = .000). A post hoc Bonferroni Wilcoxon signed rank test also found the k-value differences between each condition pair to be significant (p = .000). In addition, there was a significant positive correlation among the participant k-values of each condition with the other conditions (S2 Table, the three listed under Measure 5 with each other). Thus, the discounting parameter decreased when the amount of (delayed) reward was larger in both absolute (“2 vs 3” > “3 vs 4”) and relative (“2 vs 3” > “2 vs 4”) terms, showing that participants were more willing to wait for the delayed reward (and thus discounted it less) when it was more attractive.

Fig 13B shows the average reaction time for each attractiveness condition. The reaction time was significantly fastest in the “2 vs 4” condition (M = 1.42, SD = 0.36), slowest in the “2 vs 3” condition (M = 1.58, SD = 0.42), and moderate in the “3 vs 4” condition (M = 1.49, SD = 0.38). A non-parametric Friedman test of differences among repeated measures was conducted and rendered a Chi-square value of 36.65 that was significant (N = 65, p = .000). A post hoc Bonferroni Wilcoxon signed rank test found that the difference in reaction time between each condition pair was significant (p < .002). These results indicate that the decision of whether to discount was more difficult when the difference between the rewards and the amount of reward was smaller. Finally, there was a significant negative relationship between the k-value and reaction time for each condition (“2 vs 3”: Pearson-r = -.404, N = 65, p = .001; “3 vs 4”: Pearson-r = -.419, p = .001; “2 vs 4”: Pearson-r = -.349, p = .004) of the Delay-Discounting Task. This indicates that the participants who discounted the delayed reward more heavily made their decisions faster. Overall, the delay-discounting results verify that the participants actually considered the picture stimuli rewarding according to the “star” categories, validating this critical component of the paradigm.

### Correlation between serial-choice behavior and other measures

To identify which, if any, phenomena were related with serial-choice behavior, we calculated the Pearson correlation coefficient between serial choice and each of the experimental tasks and psychological metrics. For serial choice, we examined both the SC score and its standard deviation (SD). For the SC SD, both extreme preferences—i.e., favorite-first (with low SC score) or favorite-last (with high SC)—necessarily had low standard deviations; while the central SC scores reflected more mixed strategies across trials, and thus higher SDs. Thus, correlations with the SC score would signal differences across low to high SC scores, while correlations with the SC SD would signal differences between mixed vs. strict strategy preferences. At the same time, since the favorite-last strategy was the most dominant for the participants, a negative relationship between the SC score and the SC SD (Pearson-r = -.238, N = 69, p = .049) was inevitable (given that the favorite-last strategy had a high SC and inherently required a low standard deviation). Thus, it was important to isolate the SC score and SD effects. Therefore, if a significant relationship was found between either the SC score or the SC SD with a given task or metric, we conducted a subsequent partial correlation to isolate and verify the potential relationship.

**Fig 14:**
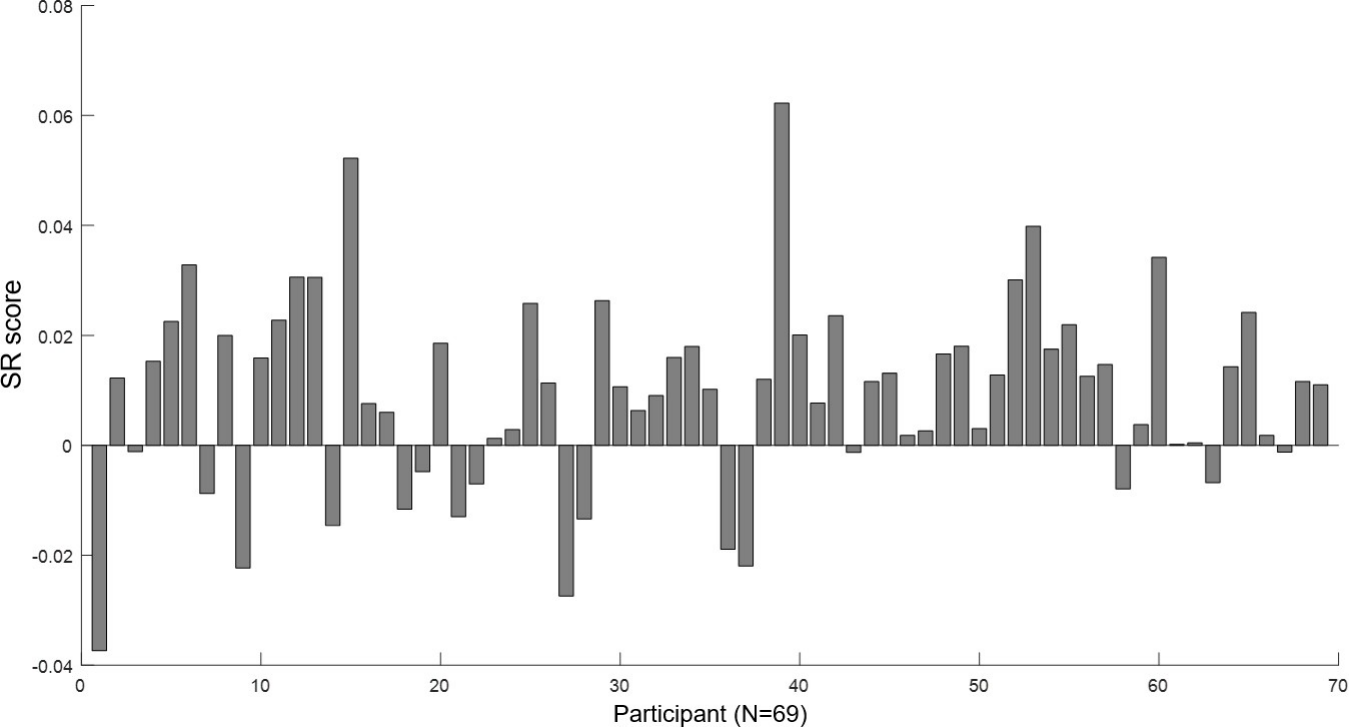
Individual results of the Sequence-Rating (SR) Task aligned with Serial-Choice (SC) score. The vertical axis represents the SR slope, i.e., the retrospective sequential experience preference; while the horizontal axis represents each participant aligned by the SC score from the Serial-Choice Task (i.e., the same participant order in Fig 6). The higher (positive) SR score reflects a preference for ascending (e.g., 1-2-3-4) sequences, while the opposite (negative) reflects that for descending (e.g., 4-3-2-1) sequences. The SC (x-axis) and SR (y-axis) scores were not correlated (Pearson-r = .133, N = 69, p = .276), and thus preferences for ascending or descending sequences did not remain consistent for individuals across the two tasks.

Delay discounting was not correlated with the SC score or SC SD (S2 Table, Measure 5). This indicates that the serial choice behavior appears to be independent of discounting future (delayed) reward, at least under our experimental conditions. Thus, for example, a favorite-first (or last) strategy did not derive from an impulsiveness stemming from steeper delay discounting. In addition, although there was a general resemblance between serial choice and sequence rating, such that most participants preferred ascending sequences, while a distinct group preferred descending, scores on the two tasks were nonetheless not correlated (Pearson-r = .133, N = 69, p = .276), nor was the SC SD (S2 Table, Measure 6). Thus, preferences for ascending or descending sequences did not remain consistent for individuals across the two tasks. Indeed, an examination of Fig 14 shows that those participants who preferred descending retrospective sequential experiences were fairly evenly distributed across SC scores, reflecting the independence of the two phenomena, at least under our experimental conditions. Moreover, no other demographic (i.e., age, education, BMI) or psychometric measures (BDI, STAI, BFI except conscientiousness and agreeableness) were significantly correlated with the SC score or the SC SD, except for three: ERQ (i.e., emotion regulation), BFI-conscientiousness, and BFI-agreeableness, described next.

**Fig 15:**
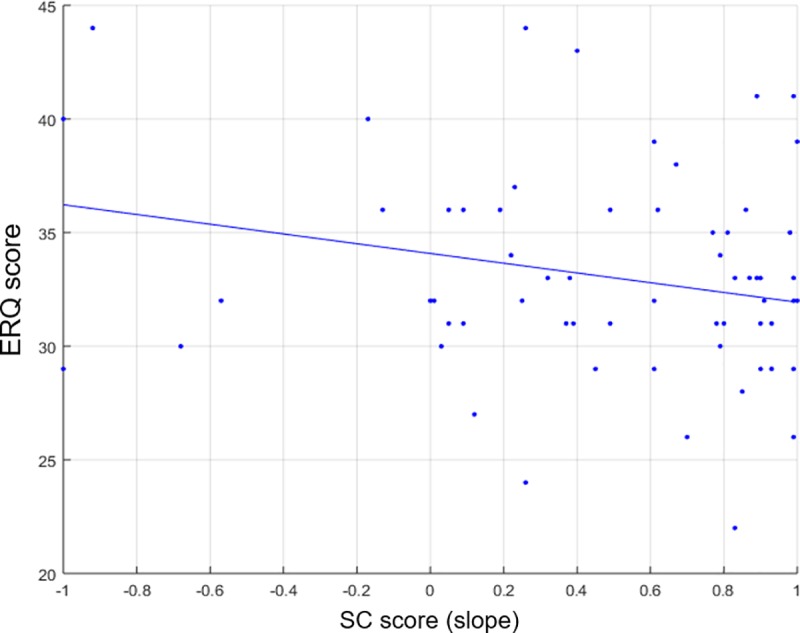
Relationship between Serial-Choice and ERQ scores. There was a negative correlation between the SC and ERQ scores, indicating that the favorite-first strategy also showed *better* emotion-regulation ability. (Pearson-r = -.254, N = 66, p = .040).

Fig 15 shows the negative relationship uncovered between the ERQ (emotion regulation) and SC scores (Pearson-r = -.254, N = 66, p = .040). Although we did not find a significant relationship between the ERQ score and SC standard deviation (Pearson-r = -.040, N = 66, p = .750), we nonetheless also conducted a partial correlation to remove any potential SD effects; as a result, the relationship between the ERQ and SC scores in fact became a bit stronger (Pearson-r = -.269, N = 66, p = .030). This negative relationship between the SC and ERQ scores suggests, perhaps counterintuitively, that those participants with a propensity to prefer a *favorite-first* strategy actually exhibited a *stronger* ability to regulate their emotions, as measured by the ERQ questionnaire.

**Fig 16:**
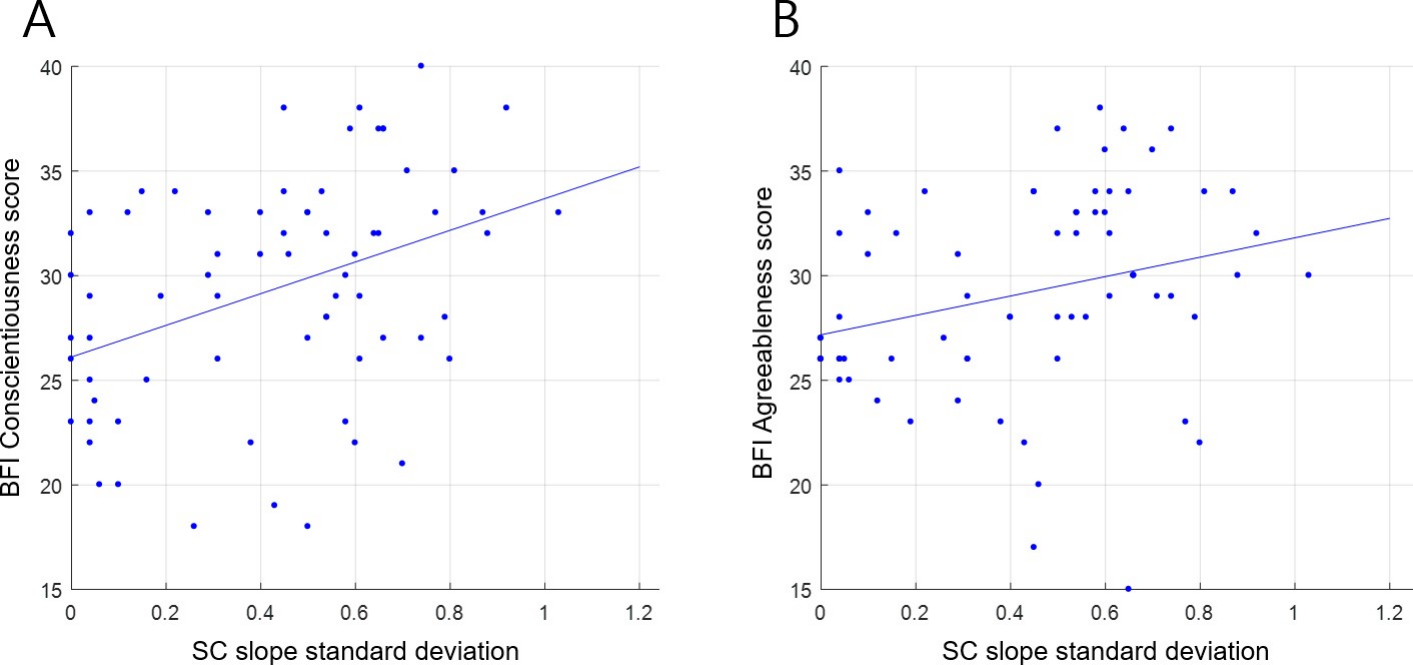
Relationship between standard deviation of Serial-Choice slope (SC SD) and BFI Conscientiousness and Agreeableness scores. We found a significant positive correlation between the standard deviation of the SC slope and the BFI Conscientiousness (15A) (Pearson-r = .390, N = 69, p = .001) and Agreeableness (15B) (Pearson-r = .269, N = 69, p = .025) scores. These results indicate that the use of more varying strategies in serial choice—compared to strict favorite-first or favorite-last strategies—was linked to more self-disciplined and considerate personality traits.

We also initially found the BFI-Conscientiousness score to have a significant negative relationship with the SC score (Pearson-r = -.239, N = 69, p = .048). However, at the same time, the BFI-Conscientiousness score was also significantly positively correlated with the standard deviation of the SC slope (Pearson-r = .390, N = 69, p = .001). Therefore, the actual relationship between the BFI-Conscientiousness score and serial choice required further clarification: i.e., whether being due to the SC score per se or the SD of the score. To determine this, we conducted partial correlation analysis controlling the effect of the SC standard deviation. As a result, we found that the relationship between the SC and BFI-Conscientiousness score (Pearson-r = -.164, p = .182) was no longer significant when the effect of the SC standard deviation was eliminated, suggesting that the BFI-Conscientiousness score was related to the SC standard deviation, not the SC score. Indeed, the relationship between the BFI-Conscientiousness score and SC SD remained significant when controlling for the SC score (Pearson-r = -.353, N = 69, p = .003). In fact, since participant age also had a marginally significant positive relationship with both BFI-Conscientiousness (Pearson-r = .216, N = 69, p = .074) and SC SD (Pearson-r = .215, N = 69, p = .076), the effect of age also needed to be controlled. Nonetheless, as a result of controlling for both SC score and age simultaneously, the relationship between BFI-Conscientiousness and SC SD again remained significant (Pearson-r = .331, N = 69, p = .006). This positive correlation between the BFI-Conscientiousness score and the SC SD shown in Fig 16A suggests that a high SC SD, which occurred by using various strategies for serial choice as opposed to the same strategy continuously, was linked to the personality traits of more self-discipline, dutifulness, and planning (versus, e.g., being more impulsive and spontaneous).

Finally, the SC SD also appeared to have a significantly positive relationship with BFI-Agreeableness (Pearson-r = .269, N = 69, p = .025). Although we did not find a significant relationship between BFI-Agreeableness and SC score (Pearson-r = -.173, N = 69, p = .154), we nonetheless applied partial correlation analysis to eliminate any possible SC score effects, and thus isolate the SC SD. As a result, the positive relationship between BFI-Agreeableness and SC SD weakened, becoming marginally significant (Pearson-r = .238, N = 69, p = .051). This marginally significant positive relationship shown in Fig 16B suggests that using multiple strategies rather than a fixed one was also potentially linked to personality traits of being considerate and compliant.

### Correlation between the other measures besides the SC score and SC standard deviation

**Fig 17:**
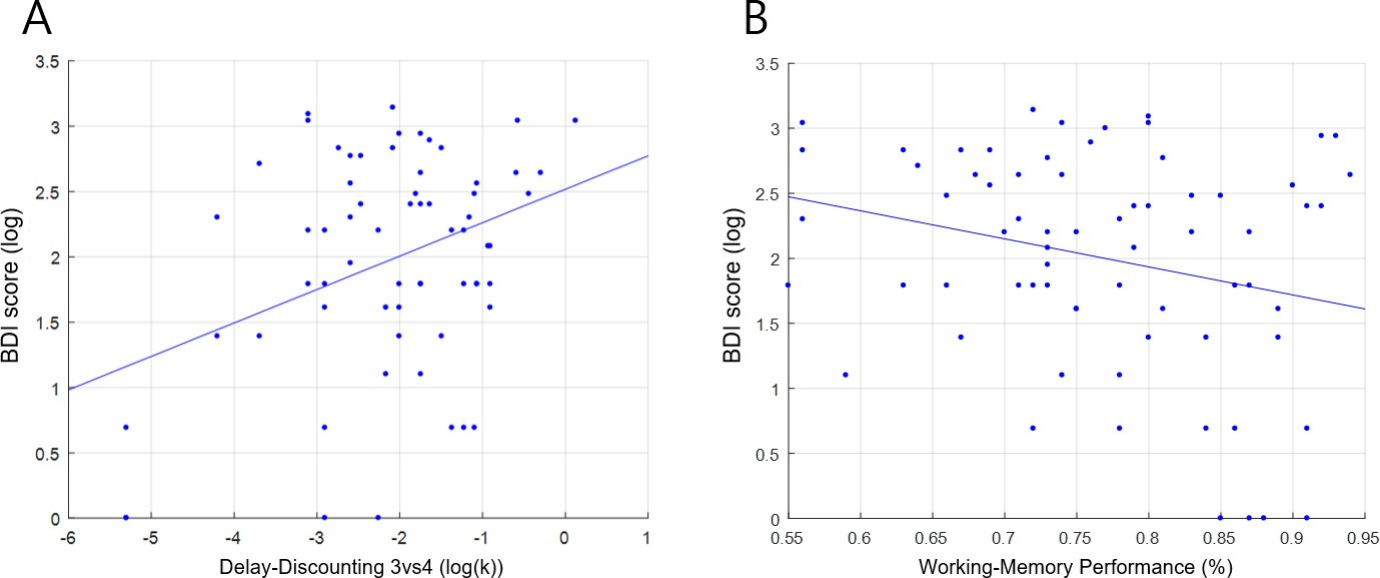
Correlation of BDI score with both delay discounting and working-memory performance. The relationships between BDI score and two behavioral task results: delay discounting parameter (A: Pearson-r = .440, N = 65, p = .000) and working-memory performance (B: Pearson-r = -.266, N = 69, p = .027). Consistent with previous studies, higher discounting of future reward and lower working-memory performance were linked to higher BDI scores, suggesting more symptoms of depression.

Finally, to provide further insight into the nature of the other tasks and metrics in the study, and at the same time, benchmark our versions of the tasks with previous research based on finding similar relationships among them, we examined the correlation coefficients of the other tasks and metrics in the experiment (i.e., besides the SC Task). First, we found a significant positive relationship between the delay-discounting parameter (k-value) and the BDI (depression) scores (Fig 17A). This relationship was significant in the “3 vs 4” (Pearson-r = .440, N = 65, p = .000) and “2 vs 4” (Pearson-r = .302, N = 65, p = .014) conditions, and thus not in the “2 vs 3” condition (Pearson-r = .033, N = 65, p = .793). These results provide evidence that our delay-discounting parameter was related with problematic psychometric symptoms, with later rewards discounted more steeply, as has been typically found with Delay-Discounting Tasks [24, 25, 26]. In addition, the k-value of the “3 vs 4” condition had a negative correlation with BFI-Openness (personality) (Pearson-r = -.299, N = 65, p = .015), which also supports the relationship between our discounting parameter and both positive and negative psychometric symptoms: discounting delayed rewards less steeply with interest in novelty and exploration, versus more steeply with withdrawing or depressive tendencies.

Second, we also found a significant negative relationship between working-memory performance and the BDI (depression) scores (Pearson-r = -.266, N = 69, p = .027) (Fig 17B). Working-memory performance also had a negative correlation with the SAI (state anxiety) scores (Pearson-r = -.275, N = 69, p = .022), and a positive relationship with BFI-Openness (Pearson-r = .294, N = 69, p = .014). These results indicate that high working-memory performance in our experimental paradigm was associated with positive psychological symptoms, and low performance with negative symptoms, consistent with previous studies [37, 38].

Third, also consistent with previous findings, we found several relationships that were significant among BFI and other psychometric data. The BFI-Neuroticism scores showed a significant positive correlation with BDI (Pearson-r = .429, N = 69, p = .000), and TAI (trait anxiety) (Pearson-r = .353, N = 69, p = .003) scores. There were also significant negative correlations between the BFI-Conscientiousness and BDI (Pearson-r = -.275, N = 69, p = .022) scores, the BFI-Extraversion and TAI (trait anxiety) scores (Pearson-r = -.445, N = 69, p = .000), and BFI-Conscientiousness with TAI (Pearson-r = -.255, N = 69, p = .034). Given that BFI-Neuroticism showed a significant negative correlation with the other BFI metrics, these results suggest that the BDI, STAI (state-trait anxiety), and BFI questionnaires indeed measured the expected symptoms of the participants [39, 40].

Fourth, besides its relationship with SC score, we also found that the ERQ (emotion regulation) scores showed a significant positive relationship with BFI-Agreeableness (Pearson-r = .406, N = 66, p = .001). This suggest a general link between greater emotion regulation and positive psychological traits.

Fifth, there was a significant positive relationship between the *picture*-*rating* scores of the Serial-Choice and Sequence-Rating tasks (Pearson-r = .380, N = 69, p = .001). This indicates there was a consistent individual tendency in ratings, such as rating all pictures relatively higher than other participants did. Comparably, there was also a significant positive relationship between the *reaction times* of the Serial-Choice and Delay-Discounting Tasks (Pearson-r = .457, N = 65, p = .000), indicating an individual tendency toward a particular reaction speed across the tasks relative to other participants.

Sixth, the age and education years of the participants significantly related with several measures. We found a positive relationship between education and the BFI-Conscientiousness score (Pearson-r = .237, N = 69, p = .050), as reported previously [41]. Age also had a significant negative relationship with number of (two-button-sequence) responses in the “3 star” category in the Effort Task (Pearson-r = -.243, N = 69, p = .044) (though not with the other ‘1, 2, or 4 star’ categories). Since there was a strongly significant positive relationship between age and education years (Pearson-r = .689, N = 69, p = .000), these relationships required further examination. After conducting partial correlation analysis, which eliminated the effect of age for the effect of education years and vice versa, none of the results remained significant. Given that age and education years were highly correlated and thus hard to discriminate, taken together, the results suggest that the older (or more educated) participants were generally more conscientious, and yet also exerted less effort to observe the rewarding pictures to some degree.

In sum, there were a number of findings among our tasks and metrics (besides serial choice) that in general validated our novel picture-rating task, as well as our methodology to carry out all tasks and questionnaires: (1) a relationship between delay discounting and both positive and negative symptoms (e.g., discounting delayed rewards less steeply when interested more in exploration, versus more steeply when withdrawn or depressed); (2) high working-memory performance associated with positive psychological symptoms and low performance with negative symptoms; (3) several relationships among BFI and other psychometric data (such as neuroticism with depression and anxiety); (4) a general link between greater emotion regulation and positive psychological traits; (5) an individual tendency toward a particular level of rating and reaction-time speed across the tasks relative to other participants; and finally, (6) older (or more educated) participants were perhaps generally more conscientious, and perhaps exerted a bit less effort than the younger (or less educated) participants in the Effort Task.

## Discussion

In the current study, we examined self-generated serial-choice behavior based on subjective preferences. We also directly investigated delay discounting, sequential experience evaluation, and working-memory performance, which have been postulated as potential underlying causes, or at least sharing the same causal influences on serial choice [1, 2, 3, 14]. To test this hypothesis, we developed a novel picture-based paradigm that enabled a direct comparison of the performance on these tasks under the same conditions. To obtain robust measures of the various behaviors and related psychological symptoms, we asked our participants to attend the study four times with an average 1-week interval. Since all of our tasks used picture stimuli with variable attractiveness levels, clarifying the participant’s specific preferences for each picture was critical. Thus, we collected rating data on a 500-picture set two separate times for each participant. The picture set was sorted by mean rating score for each participant, then divided into four (1 star ~ 4 star) groups with distinct preference levels. The consistency of the ratings in the Picture-Rating Task, as well as the results in both the Effort and Delay-Discounting Tasks, all verified the rewarding effect of the picture stimuli, and clear distinction across all attractiveness categories.

In the Serial-Choice Task, which measured self-generated serial-choice behavior, we found two dominant strategies, consistent with our previous study [1]. However, the most popular strategy in the current experiment was choosing in the opposite order of preference (from 1 star to 4 star), i.e., favorite-last, which was the second most popular strategy in the “sushi problem” [1], with the second most being favorite-first, i.e., choosing from 4 star to 1 star. Thus, the two strategies flipped in prevalence compared to the “sushi problem”. Possible reasons for this difference between studies include gender, stimuli, or procedural differences. Since we only recruited male participants for the current study, whereas the previous study included both, this difference in strategy prevalence may have resulted from gender differences. Indeed, for the “sushi problem”, the favorite-first strategy was found to be far more dominant in the female subject group, while the difference between the two dominant strategies was much less clear for the males. Regarding the experimental stimuli, we used picture stimuli versus food (sushi) to mitigate the satiation effect of the reward, and to enable a more straightforward and controlled comparison across tasks. And there is indeed substantial evidence that the different stimuli could produce different behavioral patterns [42, 43], which also could relate to the gender difference [44, 45]. Finally, other procedural differences are of course possible factors. Thus, the reasons behind the differences in the prevalence of the top two strategies (favorite-first or -last) requires further examination.

In any case, these two opposite behavioral patterns were clearly the most prevalent, with favorite-last most dominant (Fig 6). Indeed, even for the middle groups, who followed various strategies across trials, the strict favorite-last (1-2-3-4) strategy was always the most preferred. Examining reaction-times, we found that the average time of the first choices for all strategies was much slower than the choices in the rest of the sequence. This indicates that the choice strategy for the entire sequence was likely pre-determined before first choice. Thus, the entire sequence of choices was viewed as a singular overarching event, as opposed to individual choice events producing a pattern that the participants were unaware of. The serial-choice paradigm, therefore, successfully captured a fundamental higher-cognitive construct of people to organize individual events into higher-order event complexes [1, 46]. At the same time, the average time of the first choices of both the favorite-first (red) and favorite-last (blue) groups was faster than the others, suggesting that even though all strategies were pre-determined, the two dominant strategies were more strongly preferred, and thus more quickly determined (Fig 10).

Since serial choice necessarily takes place across time and actions, multiple factors could underlie the behavior, including valuation processes per se, working memory, time processing and discounting. A comparison to performance on the other tasks, then, was also examined to help delineate these effects.

Like previous research, we obtained clear discounting of delayed reward in our Delay-Discounting Task with pictures of attractiveness; and the discounting effect was mitigated with a larger delayed reward whether in absolute (“2 vs. 3” > “3 vs. 4”) or relative (“3 vs. 4” > “2 vs. 4”) terms [26, 47]. In addition, the discounting parameter had a positive relationship with the BDI (depression) score (Fig 17A), which is also consistent with previous studies [25]. Importantly, however, we did not find a significant correlation between the discounting parameter and the SC score. This indicates that, unlike what may seem intuitive on the surface, under our test conditions, time effects did not appear to significantly underlie the serial-choice preferences. Thus, for example, the favorite-first strategy did not appear to reflect an individual’s degree of impulsiveness or impatience with reward delay.

For the Sequence-Rating Task, we also corroborated other findings that have shown a general preference for ascending sequences (i.e., positive SR score), and thus, sequences that end well [14, 15, 48, 49]. Yet at the same time, a certain proportion of participants (N = 17) exhibited the opposite preference (i.e., for descending sequential experiences). Indeed, at least generally, the distributions of preferences in the SC and SR task appear similar (Figs 6 and 11A), with both reflecting a prevalent preference for ascending sequences and yet a distinct group preferring descending sequences. This general similarity suggests commonalities in the mechanisms underlying sequential processing, i.e., whether via choice or experiential ratings, with sufficient complexity that yields a broad cross section of preferences across individuals. Critically, however, as seen in Fig 14, these common general distributions did not reflect consistent preferences of individuals across the two tasks; and thus we did not find a correlation between the Serial-Choice and Sequence-Rating Tasks. Thus, for instance, a person with a strong favorite-first strategy for serial choice could yet also have a strong ascending preference for sequential experiences (with their favorite last). This lack of correlation due to the inconsistency of individual preferences across the two tasks suggests that the specific mechanisms underlying preferences differ in the two tasks (i.e., whether different set of factors and/or relative weighting of shared ones).

For example, given that sequence ratings occur after experiencing the sequence, working-memory effects (in particular, primacy for descending preference, and recency for ascending) may be stronger than for serial choice. Yet although memory performance in our study was significantly higher when the target picture was located in the last position of the sequence (Fig 12), there was not a significant correlation between working-memory performance and the sequence ratings (SR score). Although our working-memory task was not the traditional serial recall, with all potential stimuli in the sequence asked to be recalled per trial, we believe it is an efficient means of testing the same basic question: the extent to which images from each position in the list can be remembered. Given enough trials, the results should be essentially the same; and we did in fact obtain the expected primacy and recency effects. Indeed, if anything, our test should have been easier, enabling a better opportunity for capturing working-memory performance, with presumably a greater likelihood of obtaining a relationship between task performances if there was an underlying relationship between the factors. Nevertheless, the potential effect of memory on sequential preference requires further examination, including via a direct manipulation of the time scale [14, 15, 48, 49].

Another potentially important factor in sequence ratings is the affective potency of experiences. Even if one’s working-memory abilities and self-control with time assessments are sufficiently strong, the actual affective experiences could yet be most salient in the first or last positions of the sequence—for the former, due to, e.g., a comparison to ‘baseline’, and the latter due to, e.g., its nearness in time. Why some individuals were more affected earlier in the sequence versus later (preferring descending as opposed to the more prevalent ascending), could be due to other factors, including, for example, attention or sustained interest/motivation across the sequence (with attention or interest waning across the sequence for the descending preferers). Additionally, exactly how the sequence items get chunked into a singular event could differ across individuals, with the first item and its affective tag being weighted most highly for some.

The preference of most participants for sequence experiences ending well could also be significantly driven by a valuation process based on the sequence items continually improving (i.e., getting better and better). This kind of 'end' bias is supported by the fact that adding a minor (but positive) reward at the end makes a sequence less preferable [3, 11], and also that the general preference for ascending sequences weakens or disappears when the timing of the rating is delayed (like one week or more) [48, 49]. However, the same evaluation process would be expected to underlie serial choice, and yet there was no correlation between the two tasks. Nonetheless, this valuation process could be occurring in both cases (serial choice and sequence rating) for those who prefer the ascending sequences in each task, with a lack of correlation across tasks being due to additional factors such as attention that cause some individuals to change preferences across the tasks.

In sum, for sequence rating, the current study did corroborate the predominant preference for sequences ending well, yet exactly why this prevailed and why some individuals preferred descending sequences remains unclear, potentially influenced (at least to some degree) by the underlying valuation process(es), memory, attention, and affective potency. Future studies that also aim to examine multiple relevant factors under the same experimental conditions are indeed necessary. Importantly, computational modeling with direct comparison of multiple models is also needed to help disentangle and delineate actual contributions. Functional imaging studies should also help to characterize the underlying mechanisms.

In any event, we did not obtain a relationship between the Sequence-Rating and Serial-Choice Task results. Although such a null result warrants further investigation, given that our results for each task were well benchmarked with previous studies, the lack of relationship suggests that retrospective evaluation of past experiences is not directly linked to the prospective serial-choice behaviors, at least in comparable conditions as ours. The working-memory performance in the Sequence-Rating Task also did not show a relationship with the Serial-Choice Task results, which also implies that a shortage or weakness of working-memory capacity was not the reason for the strategy preferences, and in particular favorite-first. Similar with delay discounting, working-memory performance is known to be related to problematic psychological symptoms like depression and anxiety, which we also found with depression and state anxiety [37, 38]. Nonetheless, even though we obtained multiple relationships among the behavioral task results and the psychological metric data, none of the tasks or metrics were found to be related to serial choice, except for emotion regulation (the ERQ questionnaire), conscientiousness, and agreeableness (the latter two from the BFI personality questionnaire). Before considering these three findings further, we first highlight the fact that our results point to sequential choice as a unique phenomenon and thus a higher-order cognitive ability in its own right, and in particular, distinct from the discounting of future events, retrospective evaluation of event sequences, and working-memory performance. Moreover, the findings further suggest that the particular strategies found for each individual may reflect a reasonably stable personal trait, not directly related to others such as impulsivity, biases in rating experienced events, or a lack of working-memory capacity.

A relationship between serial choice and emotion regulation, on the surface, is not surprising, however, the direction is: that those with a lower SC score, and thus especially those preferring favorite-first, were more successful with emotion regulation—meaning as well that those with a higher SC score, and especially preferring their favorite last, were weaker with emotion regulation. This finding thus implies that people with a favorite-first preference tend to deal better with their emotional experiences, and if anything, are even more self-disciplined than those who prefer their favorite last. A possible explanation may entail focusing on the less-preferred rather than the most-preferred stimuli. That is, the 1-star pictures were a relatively negative event in our paradigm, and thus may have generated mild aversion. Perhaps those with a favorite-last strategy held this strategy preference to some degree as a means to tolerate these images by knowing that better was yet to come. In contrast, those with the propensity to control their emotions better could perhaps more readily tolerate the lower categories, and thus be less affected by them even with nothing better remaining in the sequence. Thus, the aversive or negative emotional responses to the lower categories may have been dampened for the favorite-first strategists [50]. From a cognitive-emotional appraisal perspective, by reappraising the situation they were in, the negative effect of emotionally evocative stimuli might be actively weakened by the favorite-first preferrers relative to favorite-last. Indeed, the ability to reappraise has been shown to be linked to greater life satisfaction, self-esteem, and well-being [31]. In our study, the positive associations (via the correlations) between emotion regulation and agreeableness, and conscientiousness and extraversion, and the inverse relationship between conscientiousness and depression, also support the notion that favorite-first behavior is a reflection of positive psychological traits, which are conducive to cognitive-emotional reappraisal. In any case, this intriguing finding of the relationship of emotion regulation to serial choice warrants further examination in future studies.

The other relationships we found with serial choice were two personality traits from the BFI questionnaire: conscientiousness and agreeableness, with the latter being marginally significant (p = .051). In both cases, these traits were related to the standard deviation of the SC score, indicating that using various strategies for serial choice rather than remaining with the same one positively correlated with the traits. Conscientiousness reflects characteristics of greater self-discipline, hard-working, dutiful, responsible, and more planning [29, 30]. Therefore, it is possible that these participants may have been more involved in the experimental context, and more considerate about what to choose. In fact, the SC-score middle groups did exhibit a longer reaction time to make their first selection than those with more extreme SC scores (Fig 10). Deeper thought about the task and sequence of choices may have also led to heightened exploration to understand the options better and find the most preferred solution and/or the avoidance of repetitive, more tedious behavior. The agreeableness personality trait might be interpreted in a similar way, with characteristics of being considerate, compliant, helpful, and concerning of others [29, 30]. These participants may be more eager to follow the instructions and perform in the experiment with more concentration and interest, which may have again led to exploring more sequence experiences, while minimizing more tedious, repetitive behavior. Moreover, variety, such as experiencing sequences that contain some less-preferred components versus sequences of only most-preferred, may also be favored by some when evaluated by retrospection [51, 52]. It will be interesting to determine if greater concentration, thoughtfulness, and the like also lead to greater pleasure in varied experiences. Indeed, exploration is also a useful strategy for finding the best and possibly optimal choice strategies, even at the risk of drifting from the best choice strategy, with the benefit of avoiding capture in local maxima. Although seeking such variety requires some cost (e.g., more cognitive energy), these personality traits may underlie why the cost is paid. In any case, our intriguing findings of conscientiousness, agreeableness (to some extent), and emotion regulation underling serial-choice behavior warrant further examination of these relationships and the possible underlying reasons for them.

The results of the current study should be treated with some caution, based on the experimental conditions and constraints. For example, again, only male participants were recruited to maximize our chances of maintaining sufficient motivation and thus reliable and stable responses throughout the experiment. Since there is evidence for gender differences with visual sexual stimuli, the results might differ with female participants [19–23, 45]. Future studies will likely have to pilot candidate related stimuli to obtain sufficient and sustained motivation with female participants. In addition, our participants were currently university undergraduates or held undergraduate degrees, in their 20s or early 30s, with no history of neurological or psychiatric abnormalities, limiting the current range of the results to this specific demographical group and psychologically “healthy” individuals. For example, people who have problematic symptoms such as impulsivity may prefer favorite-first behavior due to the lack of patience, which may then lead them to feelings of regret afterwards [6, 53]. Moreover, the length of sequence is likely to be influential in subsequent effects obtained. We chose fairly short sequence lengths in our study to simulate these types of real-world settings, as done in the “sushi problem” study [1], as well as enable a feasible overall session length to examine all the tasks together. Nonetheless, the relationship between one’s serial-choice behavior and sequence rating or working-memory performance could be different in longer sequence paradigms, for example. In the real world, to be sure, many of our serial choice sequences and remembered experiences often take longer than the timeframe we examined, let alone the uncertainty that typically exists, as well as the variety of rewards we pursue [6, 15, 52]. Future work is therefore necessary to verify and extend these findings to other realistic conditions and contexts, including with more variety in the reward types and population composition.

Nevertheless, our results match other research findings obtained under quite different paradigms in multiple instances. While at the same time, our results suggest that sequential choice may be a unique higher-order cognitive ability—separable from delay discounting, sequence rating, and basic working memory constraints—enabling us to make strategic patterns of choices in our complex, dynamic, yet sufficiently stable world; one that provides benefits to those who can plan entire action policies across longer sequences of time. Here we found evidence that certain particular cognitive-emotional and personality characteristics influence serial-choice strategy selection. Exactly how and why this occurs, as well as the driving forces underlying other serial-choice strategies, remain open questions.

## Supporting information

S1 Table Mean number of pictures for each picture set rating (‘N star’). (TIF)

S2 Table Descriptive statistics (mean and SD) and Pearson inter-correlations between measures. Measures 1–12 are depicted in both rows and columns. SC: Serial-Choice Task, DD: Delay-Discounting Task (k-value), SR: Sequence-Rating Task, WM: Working-Memory performance, EF: Effort Task, BDI: Beck Depression Inventory, SAI/TAI: State-Trait Anxiety Inventory, ERQ: Emotion Regulation Questionnaire, BFI: Big Five Inventory. For DD, N = 65, ERQ N = 66, otherwise N = 69. For Measure 5 (DD), 8 (EF), 9 (BDI), and 10 (SAI/TAI), the data were log transformed. “*” is p < .05, “**” is p < .01, “***” is p < .001 and “‘“ denotes p value between .05 ~ .10. (2-tailed)
(PNG)

S1 Fig Individual results of the Serial-Choice (SC) task: Standard deviation of the SC score. The vertical axis represents the standard deviation (SD) of the SC score (slope). The horizontal axis represents each participant, aligned by the SC score (same with Fig 6). The higher SD indicates that more varied strategies were used in the SC task across trials. Both left and right extremes of the distribution (i.e., participants who maintained strict favorite-first or favorite-last strategies) had lower SDs than the middle, indicating that the middle participants used more varied serial-choice strategies.
(TIF)

S2 Fig Rate of choices in serial-choice task. More details of the SC Task results. Panel A displays each possible strategy (N = 24) in the SC Task. Choice Strategy 1 is strict favorite-last strategy (1-2-3-4), while the 24^th^ is the opposite, favorite-first (4-3-2-1). Panel B shows the rate of choice strategies for all participants. Choice Strategy 1 (favorite-last) was most dominant, followed by Choice strategy 24 (favorite-first). Panel C shows the rate of choice strategies divided into seven subgroups. The criteria of group classification were the same as with Figs 8 and 9. The 1^st^ group had a strong preference for Choice Strategy 24 (favorite-first), while the 4^th^ ~ 7^th^ groups preferred Strategy 1 (favorite-last). The 2^nd^ and 3^rd^ groups used various strategies, although Strategy 1 was still most preferred.
(TIF)
