# Supplementary figures and images for: Preference-based serial decisions are counterintuitively influenced by emotion regulation and conscientiousness
Source: PLoS One. 2019 Oct 4;14(10):e0222797. doi: 10.1371/journal.pone.0222797 (PMC6777784; doi:10.1371/journal.pone.0222797)

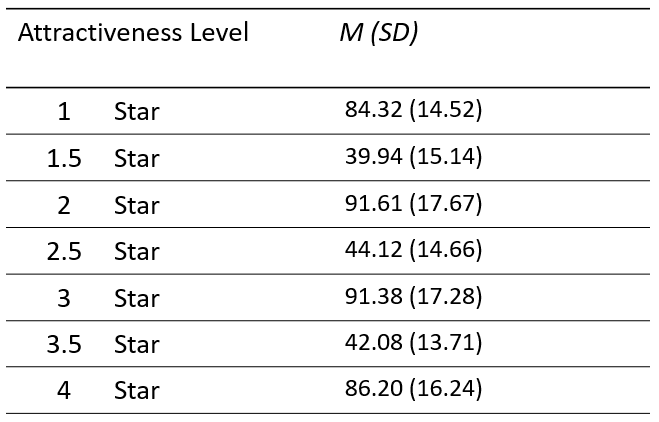

Supplement: S1 Table — (TIF) [file pone.0222797.s001.tif]

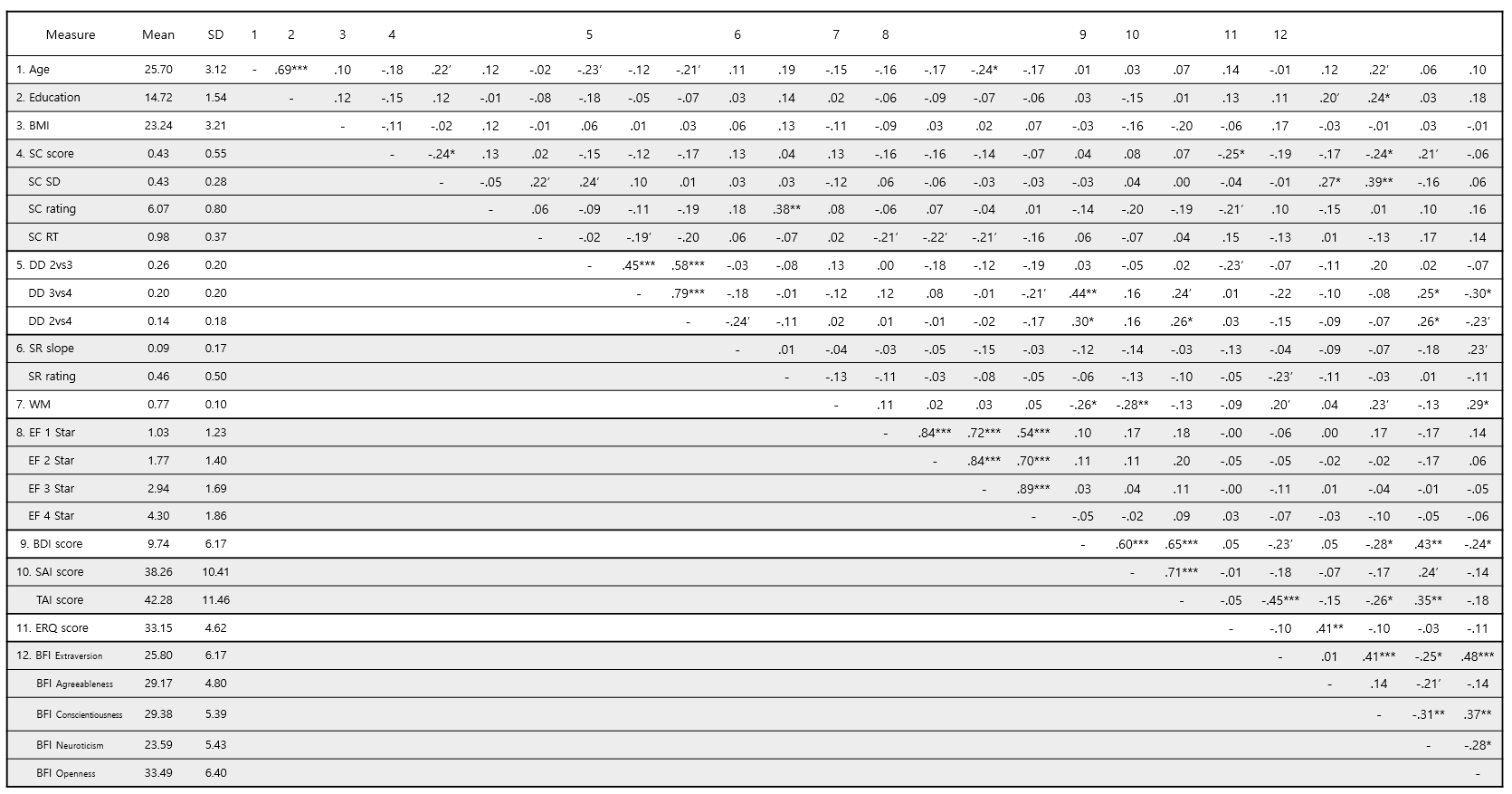

Supplement: S2 Table — Measures 1–12 are depicted in both rows and columns. SC: Serial-Choice Task, DD: Delay-Discounting Task (k-value), SR: Sequence-Rating Task, WM: Working-Memory performance, EF: Effort Task, BDI: Beck Depression Inventory, SAI/TAI: State-Trait Anxiety Inventory, ERQ: Emotion Regulation Questionnaire, BFI: Big Five Inventory. For DD, N = 65, ERQ N = 66, otherwise N = 69. For Measure 5 (DD), 8 (EF), 9 (BDI), and 10 (SAI/TAI), the data were log transformed. “*” is p < .05, “**” is p < .01, “***” is p < .001 and “‘“ denotes p value between .05 ~ .10. (2-tailed) (PNG) [file pone.0222797.s002.PNG]

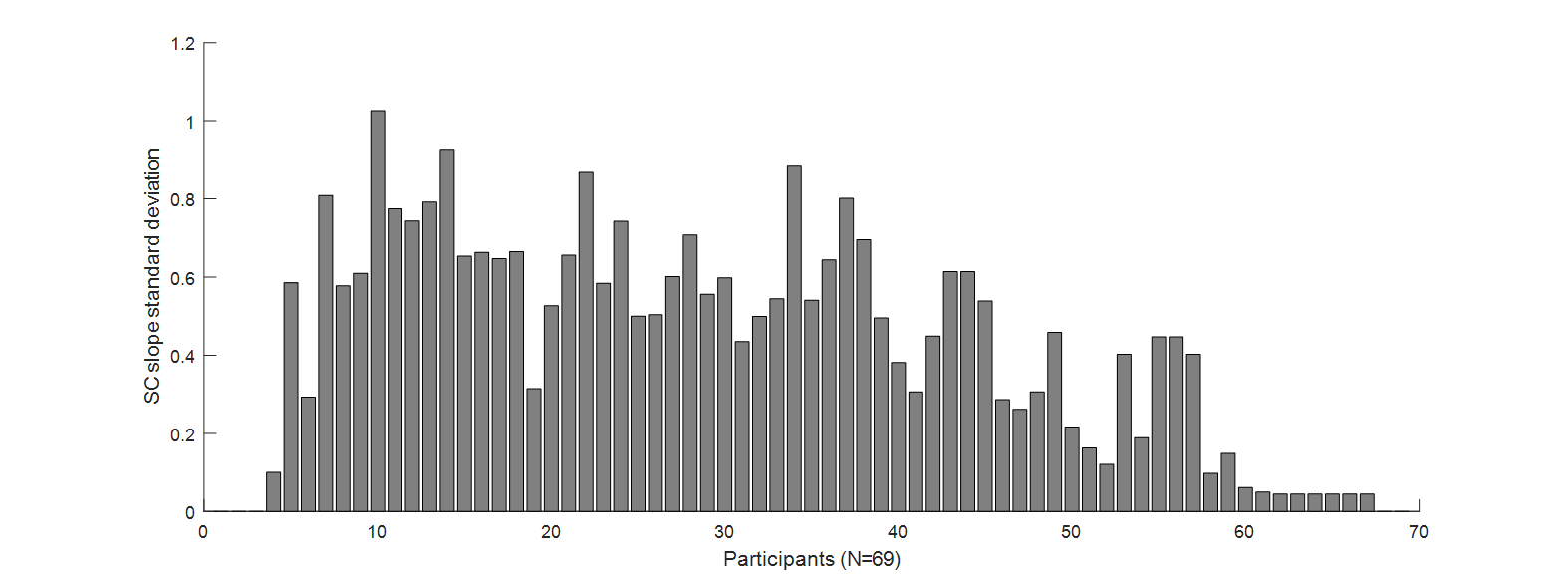

Supplement: S1 Fig — The vertical axis represents the standard deviation (SD) of the SC score (slope). The horizontal axis represents each participant, aligned by the SC score (same with Fig 6). The higher SD indicates that more varied strategies were used in the SC task across trials. Both left and right extremes of the distribution (i.e., participants who maintained strict favorite-first or favorite-last strategies) had lower SDs than the middle, indicating that the middle participants used more varied serial-choice strategies. (TIF) [file pone.0222797.s003.tif]

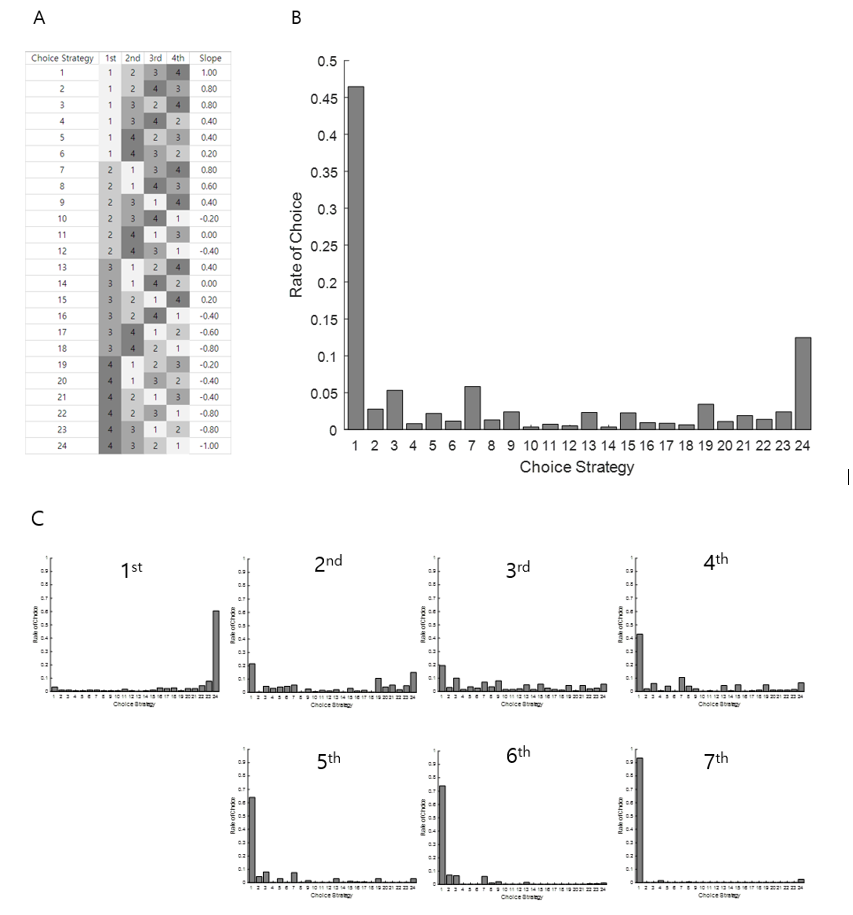

Supplement: S2 Fig — More details of the SC Task results. Panel A displays each possible strategy (N = 24) in the SC Task. Choice Strategy 1 is strict favorite-last strategy (1-2-3-4), while the 24th is the opposite, favorite-first (4-3-2-1). Panel B shows the rate of choice strategies for all participants. Choice Strategy 1 (favorite-last) was most dominant, followed by Choice strategy 24 (favorite-first). Panel C shows the rate of choice strategies divided into seven subgroups. The criteria of group classification were the same as with Figs 8 and 9. The 1st group had a strong preference for Choice Strategy 24 (favorite-first), while the 4th ~ 7th groups preferred Strategy 1 (favorite-last). The 2nd and 3rd groups used various strategies, although Strategy 1 was still most preferred. (TIF) [file pone.0222797.s004.tif]
